# iASeq: integrative analysis of allele-specificity of protein-DNA interactions in multiple ChIP-seq datasets

**DOI:** 10.1186/1471-2164-13-681

**Published:** 2012-11-29

**Authors:** Yingying Wei, Xia Li, Qian-fei Wang, Hongkai Ji

**Affiliations:** 1 Department of Biostatistics, Johns Hopkins University Bloomberg School of Public Health, 615 North Wolfe StreetBaltimore, Maryland 21205, USA; 2 CAS Key Laboratory of Genome Sciences and Information, Beijing Institute of Genomics, Chinese Academy of Sciences, Beijing 100029, P.R. China; 3 University of Chinese Academy of Sciences, Beijing 100049, P.R. China

**Keywords:** Allele-specific binding, Transcription factor, Histone modification, Data integration, Next-generation sequencing, Statistical model

## Abstract

**Background:**

ChIP-seq provides new opportunities to study allele-specific protein-DNA binding (ASB). However, detecting allelic imbalance from a single ChIP-seq dataset often has low statistical power since only sequence reads mapped to heterozygote SNPs are informative for discriminating two alleles.

**Results:**

We develop a new method iASeq to address this issue by jointly analyzing multiple ChIP-seq datasets. iASeq uses a Bayesian hierarchical mixture model to learn correlation patterns of allele-specificity among multiple proteins. Using the discovered correlation patterns, the model allows one to borrow information across datasets to improve detection of allelic imbalance. Application of iASeq to 77 ChIP-seq samples from 40 ENCODE datasets and 1 genomic DNA sample in GM12878 cells reveals that allele-specificity of multiple proteins are highly correlated, and demonstrates the ability of iASeq to improve allelic inference compared to analyzing each individual dataset separately.

**Conclusions:**

iASeq illustrates the value of integrating multiple datasets in the allele-specificity inference and offers a new tool to better analyze ASB.

## Background

In a diploid organism, each somatic cell has two copies of the genome. At certain genomic loci, gene expression, DNA methylation, transcription factor (TF) binding or histone modification (HM) can be allele-specific. In other words, the two alleles can behave differently. These phenomena, also known as allele-specific expression (ASE), allele-specific DNA methylation (ASM) and allele-specific binding (ASB, including both allele-specific TF binding and allele-specific histone modifications), can contribute to phenotypic diversity and may play important roles in adaptive evolution [1-3]. Many allele-specific (AS) events have been found to correlate with variants in genomic sequences [4-11]. Comprehensively characterizing allele-specificity therefore can help with linking genotypes to phenotypes. Abnormal AS events have also been linked to various diseases [12-15]. For instance, loss of imprinting in IGF2 has been associated with increased risk of colorectal cancer [12]. This again highlights the importance of studying allele-specificity.

Early methods for analyzing AS events rely on low-throughput technologies such as real time quantitative PCR [1]. Later, application of SNP arrays has made the AS analysis high-throughput [16-19]. More recently, the rapidly evolving high-throughput sequencing technologies opened the door to produce digital read-out of AS events genome-wide without being constrained by any specific array design [5,14,15,20-24]. This brings many new opportunities as well as analytical challenges.

ChIP-seq, a technology that couples chromatin immunoprecipitation with high-throughput sequencing, has become the state-of-the-art approach for mapping genome-wide TF binding sites and HMs [25-28]. However, so far the value of this technology for studying ASB has not been fully utilized. Detecting ASB from a single ChIP-seq dataset often suffers from low statistical power. This is because only a small fraction of reads in each ChIP-seq sample are mapped to heterozygote SNPs, and only these reads are informative for inferring allele-specificity. To make the ChIP-seq based ASB analysis more useful, it is important to have either experimental or analytical innovations to increase the power for detecting allele-specificity.

ChIP-seq data in public domains grow rapidly. A recently developed database hmChIP, for instance, has compiled over 450 human and mouse ChIP-seq datasets representing approximately 2000 samples from 140+ different TFs and HMs [29,30]. The large volume of data provides a new opportunity to improve detection of ASB. Conceptually, an integrative analysis of ChIP-seq data for different TFs and HMs from the same individual and cell type may allow one to discover the synergistic correlation patterns of allele-specificity among different proteins. These correlation patterns can then be utilized to integrate information from multiple datasets to improve the ASB detection. For example, if the allelic imbalance of TF A and HM B always co-occur, then analyzing their ChIP-seq data jointly will increase the effective number of reads available for allelic inference which will then increase the statistical power. Unfortunately, existing data analysis tools cannot deal with this emerging opportunity. Methods available for analyzing ASE or ASB using the next-generation sequencing data are all designed for analyzing one dataset at a time. While a few methods are developed for solving problems such as read mapping biases [31], construction of individualized genome sequences [32], and combining multiple SNPs in the same gene to infer ASE [33], no methods and software tools are available for jointly analyzing multiple ChIP-seq datasets together to discover synergy patterns of allele-specificity among multiple proteins and then use the correlation patterns to increase the power of ASB detection by borrowing information across datasets.

In this article, we present an integrated solution to this problem by developing a new approach, iASeq, for jointly analyzing allele-specificity in multiple ChIP-seq datasets. iASeq uses a Bayesian hierarchical mixture model to describe unknown correlation patterns of allele-specificity among multiple datasets. These patterns can be discovered automatically from the data by fitting the model using an Expectation-Maximization (EM) algorithm. Using the identified correlation patterns, the model allows one to integrate information from multiple datasets to improve the ASB detection. Applying this approach, we analyzed 40 ENCODE [34] ChIP-seq datasets in GM12878 cells, representing a total of 77 samples from 34 TFs and HMs. The analysis demonstrates the ability of iASeq to automatically integrate information from multiple datasets to significantly improve the detection of allelic imbalance. iASeq is implemented as an R package which is freely available from Bioconductor [35].

## Methods

### Data structure

**Figure 1:**
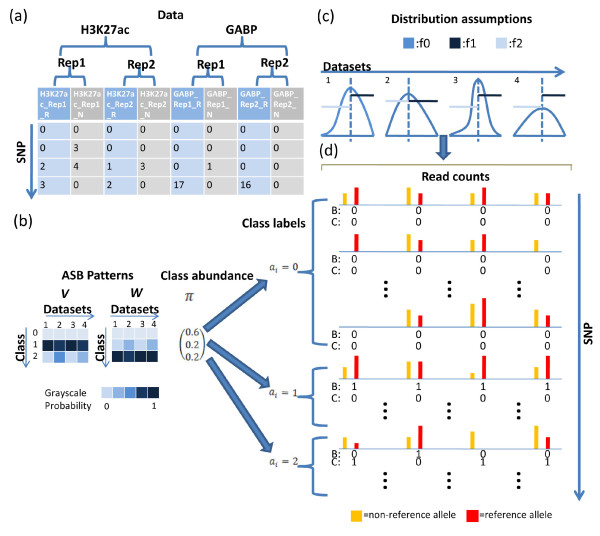
**The iASeq model. (a)** An example of the data structure. Each row represents a SNP and each column corresponds to either the reference allele (R) or the non-reference allele (N) read counts from a ChIP-seq sample in a dataset. A dataset could be a TF ChIP-seq experiment or a HM ChIP-seq experiment, and can have multiple replicate samples (Rep). iASeq assumes the following data generating process. **(b)** First, SNPs belong to *K* + 1 classes with different ASB patterns. For each SNP, a class label *a*_*i*_is randomly assigned according to a class abundance probability vector ***Π***. Given the class label, a configuration [*b*_*id*_,*c*_*id*_] is generated for each SNP in each dataset according to the probabilistic allele-specificity patterns specified by two vectors ***V***_*k*_and ***W***_*k*_. In the figure, the darkness of each cell in ***V*** and ***W*** represents the probability for *b*_*id*_or *c*_*id*_to be 1. **(c)** Next, a skewing probability *p*_*idj*_is generated for each SNP *i*, dataset *d* and replicate sample *j* based on [*b*_*id*_,*c*_*id*_]. The distribution of *p*_*idj*_for NS SNPs in each sample follows a Beta distribution (blue lines). *p*_*idj*_s for SR SNPs are uniformly distributed in the interval [*p*_*dj*0_,1] where *p*_*dj*0_is the mean of the background Beta distribution (dark blue lines). *p*_*idj*_s for SN SNPs are uniformly distributed in the interval [0,*p*_*dj*0_] (light blue lines). **(d)** Finally, given the configuration [*b*_*id*_,*c*_*id*_], skewing probability *p*_*idj*_and a total read count *n*_*idj*_for SNP *i*, dataset *d* and sample *j*, the read count for each allele is generated according to a binomial distribution. The length of the orange bar represents the non-reference allele read count, and the length of the red bar represents the reference allele read count.

Suppose there are *D* ChIP-seq datasets generated using cells from the same individual and the same cell type. Each dataset *d* corresponds to one TF or HM, and has *J*_*d*_ replicate samples (Figure 1a). Different datasets represent different TFs or HMs, or data generated by different labs. For the individual in question, assume one is interested in analyzing *I* heterozygote SNPs with known genotypes. We want to know whether the two alleles of each SNP behave differently in each dataset, and if possible how the AS events are correlated among datasets. For each SNP, the allele consistent with the reference genome is called the *reference allele*, and the other allele is called the *non-reference allele*.

After read mapping and data preprocessing (see Additional file 1: Supplemental Methods S.1), we count reads for each allele at each heterozygote SNP. For SNP *i*, dataset *d* and replicate sample *j*, let *x*_*idj*_ and *y*_*idj*_be the read counts for the reference allele and non-reference allele respectively. Let *n*_*idj*_=*x*_*idj*_ + *y*_*idj*_ be the total read count (See Figure 1a for a toy example). Protein-DNA binding can be skewed to the reference allele (SR), skewed to the non-reference allele (SN), or not allele-specific (NS). We use a binary variable *b*_*id*_ to indicate whether SNP *i* is SR (*b*_*id*_=1) or not (*b*_*id*_=0) in dataset *d*. If *b*_*id*_=1, then SNP *i* is assumed to be SR in all replicate samples in dataset *d*. Similarly, we introduce another binary indicator *c*_*id*_ to indicate whether SNP *i* is SN or not in dataset *d*. *b*_*id*_and *c*_*id*_ cannot be equal to one at the same time. If *b*_*id*_=0 and *c*_*id*_=0, then SNP *i* is NS in dataset *d*. The configuration at each SNP *i* can be described by two vectors ***B***_*i*_=(*b*_*i*1_,⋯,*b*_*iD*_) and ***C***_*i*_=(*c*_*i*1_,⋯,*c*_*iD*_) (See Figure 1d for a cartoon illustration). Based on these notations, (*x*_*idj*_,*y*_*idj*_), or equivalently (*x*_*idj*_,*n*_*idj*_), are the observed data for SNP *i* in sample (*d,j*), whereas the indicators *b*_*id*_ and *c*_*id*_ are unobserved.

### Main intuition and challenge

Our primary goal is to infer for each SNP whether there is allelic imbalance in each dataset. This is equivalent to inferring *b*_*id*_ and *c*_*id*_. A simple solution to this problem is to analyze each individual dataset separately, but this approach has low statistical power since the counts (*x*_*idj*_,*n*_*idj*_) usually are small.

If one knows how different datasets are correlated in terms of allelic imbalance, this knowledge may be used to improve the data analysis. For instance, if the allelic imbalance of two proteins A and B are closely correlated, then observing skewed read counts for protein A will provide information for inferring the allelic imbalance of protein B. Integrating the data from both A and B will increase the effective number of reads available for statistical inference, which will then lead to increased statistical power.

In reality, how different proteins are correlated is usually unknown. However, one may learn it by studying the data from many SNPs. Each SNP has three possible states in each dataset: SR, SN and NS. For *D* datasets, there are 3^*D*^ possible configurations in total. From studying many SNPs, one can know the relative frequencies (or mixing proportions) of these 3^*D*^configurations. The mixing proportions will tell how different datasets are correlated. For instance, let [*s*_1_,*s*_2_,⋯,*s*_*D*_] be the skewness configuration of a SNP in the *D* datasets. If the mixing proportions for three configurations [*NS*,*NS*,⋯,*NS*], [*SR*,*SR*,⋯,*SR*] and [*SN*,*SN*,⋯,*SN*] are 0.9, 0.05 and 0.05, then no other configurations exist in the data and all datasets are perfectly correlated in terms of the allelic imbalance. In other words, at a particular SNP, if one dataset is SR, then all the other datasets are also SR. If one is SN, then all the others are also SN. On the other hand, if other configurations have non-zero mixing proportions, then not all datasets are perfectly correlated, and at a particular SNP, one allows the possibility that only a subset of datasets are correlated. For instance, if the mixing proportion for a configuration [*SR*,*SR*,*NS*,⋯,*NS*] is 0.03, then there will be 3% of SNPs that are skewed to the reference allele in the first two datasets but not skewed in the other datasets. Therefore, knowing the mixing proportions of all 3^*D*^configurations will tell one the correlation structure in the data. This knowledge can then be used to improve statistical inference at each individual SNP by facilitating information sharing across datasets. For example, if the configuration [*SR, SR, SN*] has a much higher mixing proportion than [*SR, SR, NS*], then observing strong skewness towards the reference allele of a SNP in the first two datasets will imply that, a priori, the SNP is highly likely to be skewed to the non-reference allele in the third dataset and has much lower probability to be non-skewed for both alleles. The principle here is the same as the principle represented by the Bayesian hierarchical models in the statistical literature.

A limitation of this approach is that one has to enumerate all 3^*D*^ AS configurations in order to describe the correlation. As the number of datasets increases, the number of possible configurations increases exponentially. Thus this approach does not scale well with the increasing *D*. Later, in our analysis of GM12878 data, *D*=40 and 3^*D*^>10^19^. This simple approach is clearly intractable.

To circumvent the difficulty of documenting the frequencies of all 3^*D*^configurations, iASeq employs a technique that can describe the major correlation patterns in the data using a few probability vectors whose values vary from 0 to 1 rather than being dichotomous (i.e., 0 or 1). This approach significantly reduces the model complexity but keeps the flexibility to account for all 3^*D*^configurations. It is easily scalable to increasing dataset number. The correlation structure in the model can then be used to improve the statistical inference of allelic imbalance at each SNP in each individual dataset.

### Probability model

iASeq is based on the Bayesian hierarchical mixture model below that uses several probability vectors to describe the major correlation patterns among multiple datasets (Figure 1). The model assumes that SNPs can be grouped into *K* + 1 classes with different allele-specificity patterns (*K*≪3^*D*^), and the observed data are viewed as generated as follows: 

● First, a class label *a*_*i*_is randomly assigned to each SNP *i* according to a probability vector ***Π***=(*Π*_0_,*Π*_1_,⋯,*Π*_*K*_). Here, *Π*_*k*_=*Pr*(*a*_*i*_=*k*) is the prior probability to assign a SNP to class *k*. $\underset{k}{\sum }\Pi_{k}=1$.

● If the class label *a*_*i*_=0, then ***B***_*i*_=(0,⋯,0) and ***C***_*i*_=(0,⋯,0). In other words, all SNPs in class 0 are background SNPs, and they are NS in all datasets. If *a*_*i*_=*k*and *k*≠0, then SNP *i* can be skewed, and its [*b*_*id*_;*c*_*id*_]s in different datasets are generated independently according to the following probabilities: *Pr*(*b*_*id*_=1,*c*_*id*_=0|*a*_*i*_=*k*)=*v*_*kd*_and *Pr*(*b*_*id*_=0,*c*_*id*_=1|*a*_*i*_=*k*)=*w*_*kd*_. We assume *v*_*kd*_ + *w*_*kd*_<1, i.e., *Pr*(*b*_*id*_=0,*c*_*id*_=0|*a*_*i*_=*k*)=1−*v*_*kd*_−*w*_*kd*_>0. The model implies that each class is associated with two vectors of probabilities ***V***_*k*_=(*v*_*k*1_,⋯,*v*_*kD*_) and ***W***_*k*_=(*w*_*k*1_,⋯,*w*_*kD*_). For SNPs in class *k*, ***B***_*i*_and ***C***_*i*_are generated according to the probabilities in ***V***_*k*_and ***W***_*k*_.

● Next, the observed read counts are generated based on the AS configurations specified by ***B***_*i*_s and ***C***_*i*_s. Consider SNP *i* and dataset *d*. If *b*_*id*_=1, then (*x*_*idj*_,*n*_*idj*_) in each replicate sample (*d,j*) is generated according to a probability distribution *Pr*(*x*_*idj*_,*n*_*idj*_|*b*_*id*_=1,*c*_*id*_=0)=*Pr*(*n*_*idj*_|*b*_*id*_=1,*c*_*id*_=0)*Pr*(*x*_*idj*_|*n*_*idj*_,*b*_*id*_=1,*c*_*id*_=0)≡*Pr*(*n*_*idj*_)*f*_*idj*1_(*x*_*idj*_). Here we assume that the marginal distribution of *n*_*idj*_does not depend on *b*_*id*_and *c*_*id*_, and we use *f*_*idj*1_(*x*_*idj*_) to denote the conditional distribution *Pr*(*x*_*idj*_|*n*_*idj*_,*b*_*id*_=1,*c*_*id*_=0). Data in different replicate samples are assumed to be generated independently. Similarly, if *c*_*id*_=1, then (*x*_*idj*_,*n*_*idj*_)s are generated according to *Pr*(*x*_*idj*_,*n*_*idj*_|*b*_*id*_=0,*c*_*id*_=1)=*Pr*(*n*_*idj*_)*f*_*idj*2_(*x*_*idj*_). If *b*_*id*_=0 and *c*_*id*_=0, then (*x*_*idj*_,*n*_*idj*_)s are generated according to *Pr*(*x*_*idj*_,*n*_*idj*_|*b*_*id*_=0,*c*_*id*_=0)=*Pr*(*n*_*idj*_)*f*_*idj*0_(*x*_*idj*_).

For SNP *i* and dataset *d*, we organize data from all replicates *j*=1,⋯,*J*_*d*_ into ***X***_*id*_=(*x*_*id*1_,⋯,*x*_*idJ*__*d*_) and ***N***_*id*_=(*n*_*id*1_,⋯,*n*_*idJ*__*d*_). For SNP *i*, ***X***_*i*_=(***X***_*i*1_,⋯,***X***_*iD*_) and ***N***_*i*_=(***N***_*i*1_,⋯,***N***_*iD*_) contain data from all datasets. The final observed data are ***X***=(***X***_1_,⋯,***X***_*I*_) and ***N***=(***N***_1_,⋯,***N***_*I*_) which are the ensemble of data from all SNPs.

Let ***A***=(*a*_1_,⋯,*a*_*I*_) be the collection of class membership indictors of all SNPs, and let ***B***=(***B***_1_,⋯,***B***_*I*_) and ***C***=(***C***_1_,⋯,***C***_*I*_) be the SR and SN indictors for all SNPs. ***A, B*** and ***C*** are the unobserved missing data one wants to infer.

Organize the probability vectors ***V***_*k*_and ***W***_*k*_ from different classes into two matrices $\left(V_{K\times D}={\left(V_{1}^{T},\cdots \;,V_{K}^{T}\right)}^{T}\right)$ and $\left(W_{K\times D}={\left(W_{1}^{T},\cdots \;,W_{K}^{T}\right)}^{T}\right)$. ***V***, ***W***, and the probability vector ***Π*** that describes the class abundance are the unknown model parameters. *K* is assumed to be fixed. The choice of *K* and specification of data generating distributions *Pr*(*n*_*idj*_), *f*_*idj*0_(*x*_*idj*_), *f*_*idj*1_(*x*_*idj*_) and *f*_*idj*2_(*x*_*idj*_) will be discussed later.

Based on this model, each SNP class *k* (*k*≠0) is associated with two vectors of probabilities ***V***_*k*_and ***W***_*k*_ which characterize the allelic imbalance preferences in different datasets for SNPs belonging to class *k*. For example, if a class has [***V***_*k*_;***W***_*k*_] = [(0.8,0.7,0.1,0.1); (0.1,0.1,0.8,0.1)], then SNPs in this class have high probability to be SR in datasets 1 and 2, and high probability to be SN in dataset 3, but they have low probability to be allele-specific in dataset 4. Since ***V***_*k*_ and ***W***_*k*_are probabilities rather than 0-1 vectors, each class *k* can generate all 3^*D*^ AS configurations. Therefore, SNPs in the same class are not required to have the same AS configuration (e.g., a class can have one SNP with configuration [*SR*,*SR*,*NS*,*NS*] while at the same time another SNP with configuration [*SR*,*NS*,*SR*,*NS*]), although they usually have similar AS configurations because SNPs in the same class are all generated using the same probability vectors. Meanwhile, there are *K* different classes, and each class has a different [***V***_*k*_;***W***_*k*_] which specifies a different preference to generate the skewing configurations. Thus, whereas SNPs in the same class tend to have similar [***B***_*i*_;***C***_*i*_] configurations, SNPs from different classes tend to have very different configurations. Conceptually, this is similar to a model-based clustering analysis in which SNPs are grouped into *K* + 1 clusters based on their [***B***_*i*_;***C***_*i*_] configurations. However, an important difference here is that [***B***_*i*_;***C***_*i*_]s are unknown.

Our model assumes that [*b*_*id*_;*c*_*id*_]s of the same SNP in different datasets are a priori independent conditional on the class membership *a*_*i*_. However, [*b*_*id*_;*c*_*id*_]s from different datasets are not independent marginally if one integrates out the class label *a*_*i*_. For example, the marginal probability *Pr*([*b*_*id*_;*c*_*id*_]= [1;0])=$\underset{k}{\sum }\mathrm{Pr}([b_{\mathrm{id}};c_{\mathrm{id}}]=\;[1;0]|a_{i}=k)\mathrm{Pr}(a_{i}=k)=$$\overset{K}{\underset{k=1}{\sum }}\Pi_{k}v_{\mathrm{kd}}$. On the other hand, the joint probability $\;\mathrm{Pr}([\;B_{i};C_{i}]=[\;(1,1,\;\cdots \;,\;1);(0,0,\cdots \;,0)])\;=\;\overset{K}{\underset{k=1}{\sum }}\Pi_{k}(\underset{d}{\prod }v_{\mathrm{kd}})$, which is clearly different from the product of the marginals $\underset{d}{\prod }\mathrm{Pr}([b_{\mathrm{id}};c_{\mathrm{id}}]=[1;0])=\underset{d}{\prod }(\overset{K}{\underset{k=1}{\sum }}\Pi_{k}v_{\mathrm{kd}})$. This explains why our model can be used to describe the correlation among multiple datasets despite the conditional independence assumption. Intuitively, if one views the model as a clustering analysis of SNPs based on [***B***_*i*_;***C***_*i*_], then each cluster will represent a co-occurrence pattern of allele-specificity across multiple proteins. The marginal correlation among multiple datasets is described by multiple clusters, whereas within each cluster the data in different datasets are generated independently. In real data, a small *K* (i.e., a small number of SNP classes) usually is sufficient to describe the major correlation structure among datasets. Using ***Π***, ***V*** and ***W*** to describe the correlation among datasets only requires *O*(*KD*) parameters, which is significantly less complex than *O*(3^*D*^) parameters. At the same time, the iASeq model still provides the flexibility to accommodate all 3^*D*^ possible [***B***_*i*_;***C***_*i*_] configurations as all of them have non-zero probability to occur.

### Data generating distributions

To fully specify the model, one also needs to specify the data generating distributions *Pr*(*x*_*idj*_,*n*_*idj*_|*b*_*id*_,*c*_*id*_)=*Pr*(*n*_*idj*_)*Pr*(*x*_*idj*_|*n*_*idj*_,*b*_*id*_,*c*_*id*_). The primary goal of iASeq is to infer whether two alleles are different. We assume that information on allele-specificity is only contained in *Pr*(*x*_*idj*_|*n*_*idj*_,*b*_*id*_,*c*_*id*_), and therefore the exact form of *Pr*(*n*_*idj*_), i.e., the marginal probability distribution of the total read count, is irrelevant for our purpose. As such, we mainly focus on modeling the conditional distribution of *x*_*idj*_given *n*_*idj*_, *b*_*id*_ and *c*_*id*_, i.e., the three distributions *f*_*idj*0_(*x*), *f*_*idj*1_(*x*) and *f*_*idj*2_(*x*).

iASeq models these distributions hierarchically in two steps. First, *x*_*idj*_ is assumed to follow a binomial distribution *x*_*idj*_|*n*_*idj*_,*p*_*idj*_∼*Bin*(*n*_*idj*_,*p*_*idj*_), where *p*_*idj*_ is the probability that a read generated at SNP *i* in sample (*d*,*j*) represents the reference allele. Next, we model *p*_*idj*_depending on the values of *b*_*id*_and *c*_*id*_.

If *b*_*id*_=0 and *c*_*id*_=0, SNP *i* is NS in dataset *d*. In this case, we assume that *p*_*idj*_follows a Beta distribution *Beta*(*α*_*dj*_*β*_*dj*_) with mean *p*_*dj*0_=*α*_*dj*_/(*α*_*dj*_ + *β*_*dj*_). Note that a simpler model for *p*_*idj*_ would be to set it to a constant *p*_*dj*0_ which reflects the background ratio of read counts between two alleles. However, previous studies have shown that many background SNPs can have *p*_*idj*_ slightly different from the average background *p*_*dj*0_even though they do not have biologically meaningful allele-specificity [33]. As a result, a constant *p*_*dj*0_ is not sufficient to describe the background variation. For this reason, we adopt the Beta distribution to describe *p*_*idj*_ instead of setting it to a constant (See the blue lines illustrated for *f*(*p*_*idj*_|*b*_*id*_=0,*c*_*id*_=0) in Figure 1c). In the ideal world, the mean of the Beta distribution, *p*_*dj*0_, would be equal to 0.5. However, in reality *p*_*dj*0_may be slightly different from 0.5 due to various sources of read mapping biases. For example, allowing the same number of mismatches, reads from the reference allele are easier to be mapped back to the reference genome than reads from the non-reference allele. Therefore, in iASeq *p*_*dj*0_may take values different from 0.5. Indeed, it is determined by the parameters *α*_*dj*_ and *β*_*dj*_ in the Beta distribution which are estimated from the data using a moment matching approach (see Additional file 1: Supplemental Method S.2). Once estimated, *α*_*dj*_, *β*_*dj*_ and *p*_*dj*0_are treated as fixed and known parameters. Based on the model for *p*_*idj*_, we integrate out all possible values of *p*_*idj*_ to obtain the distribution of *x*_*idj*_conditional on *b*_*id*_=0 and *c*_*id*_=0, which is a beta-binomial distribution:

(1) $$\begin{array}{lll} \;f_{\mathrm{idj}0}(x_{\mathrm{idj}}) & =\mathrm{Pr}\left(x_{\mathrm{idj}}|n_{\mathrm{idj}},b_{\mathrm{id}}=0,c_{\mathrm{id}}=0\right)\; & \;\\ & =\int_{0}^{1}\mathrm{Pr}(x_{\mathrm{idj}}|n_{\mathrm{idj}},p_{\mathrm{idj}},b_{\mathrm{id}}=0,c_{\mathrm{id}}=0)\; & \;\\ & \;\times f\left(p_{\mathrm{idj}}|b_{\mathrm{id}}=0,c_{\mathrm{id}}=0\right)dp_{\mathrm{idj}}\; & \;\\ & =\frac{C_{n_{\mathrm{idj}}}^{x_{\mathrm{idj}}}}{B\left(\alpha_{\mathrm{dj}},\beta_{\mathrm{dj}}\right)}\;\int_{0}^{1}\;\;p^{x_{\mathrm{idj}}\;+\;\alpha_{\mathrm{dj}}-1}{\left(1\;-\;p\right)}^{n_{\mathrm{idj}}-x_{\mathrm{idj}}+\beta_{\mathrm{dj}}-1}\;\mathrm{dp}\; & \;\\ & =\frac{C_{n_{\mathrm{idj}}}^{x_{\mathrm{idj}}}B\left(x_{\mathrm{idj}}+\alpha_{\mathrm{dj}},n_{\mathrm{idj}}-x_{\mathrm{idj}}+\beta_{\mathrm{dj}}\right)}{B\left(\alpha_{\mathrm{dj}},\beta_{\mathrm{dj}}\right)}\; \end{array}$$

Here $\left(C_{n}^{k}\right)$ is the binomial coefficients “*n* choose *k*”, and *B*(*..*) is the beta function.

If *b*_*id*_=1 and *c*_*id*_=0, SNP *i* is SR in dataset *d*. In this case, we assume that *p*_*idj*_ follows a uniform distribution *U*[*p*_*dj*0_,1](See the dark blue lines illustrated for *f*(*p*_*idj*_|*b*_*id*_=1,*c*_*id*_=0) in Figure 1c). Here *p*_*dj*0_=*α*_*dj*_/(*α*_*dj*_ + *β*_*dj*_) is defined as above. After integrating out *p*_*idj*_, the distribution of *x*_*idj*_conditional on *b*_*id*_=1 and *c*_*id*_=0 is

(2) $$\begin{array}{lll} \;f_{\mathrm{idj}1}(x_{\mathrm{idj}}) & =\mathrm{Pr}(x_{\mathrm{idj}}|n_{\mathrm{idj}},b_{\mathrm{id}}=1,c_{\mathrm{id}}=0)\; & \;\\ & =\int_{0}^{1}\mathrm{Pr}(x_{\mathrm{idj}}|n_{\mathrm{idj}},p_{\mathrm{idj}},b_{\mathrm{id}}=1,c_{\mathrm{id}}=0)\; & \;\\ & \;\times f(p_{\mathrm{idj}}|b_{\mathrm{id}}=1,c_{\mathrm{id}}=0)dp_{\mathrm{idj}}\; & \;\\ & =\frac{C_{n_{\mathrm{idj}}}^{x_{\mathrm{idj}}}}{1-p_{\mathrm{dj}0}}\int_{p_{\mathrm{dj}0}}^{1}p^{x_{\mathrm{idj}}}{\left(1-p\right)}^{n_{\mathrm{idj}}-x_{\mathrm{idj}}}\mathrm{dp}\; \end{array}$$

If *b*_*id*_=0 and *c*_*id*_=1, SNP *i* is SN in dataset *d*, and we assume that *p*_*idj*_ follows a uniform distribution *U*[0,*p*_*dj*0_] (See the light blue lines illustrated for *f*(*p*_*idj*_|*b*_*id*_=0,*c*_*id*_=1) in Figure 1c). After integrating out *p*_*idj*_, the distribution of *x*_*idj*_conditional on *b*_*id*_=0 and *c*_*id*_=1 is

(3) $$\begin{array}{lll} \;f_{\mathrm{idj}2}(x_{\mathrm{idj}}) & =\mathrm{Pr}(x_{\mathrm{idj}}|n_{\mathrm{idj}},b_{\mathrm{id}}=0,c_{\mathrm{id}}=1)\; & \;\\ & =\int_{0}^{1}f(x_{\mathrm{idj}}|n_{\mathrm{idj}},p_{\mathrm{idj}},b_{\mathrm{id}}=0,c_{\mathrm{id}}=1)\; & \;\\ & \;\times f(p_{\mathrm{idj}}|b_{\mathrm{id}}=0,c_{\mathrm{id}}=1)dp_{\mathrm{idj}}\; & \;\\ & =\frac{C_{n_{\mathrm{idj}}}^{x_{\mathrm{idj}}}}{p_{\mathrm{dj}0}}\int_{0}^{p_{\mathrm{dj}0}}p^{x_{\mathrm{idj}}}{\left(1-p\right)}^{n_{\mathrm{idj}}-x_{\mathrm{idj}}}\mathrm{dp}\; \end{array}$$

### Joint probabilities and model fitting

Based on the model above, the complete data likelihood can be derived as:

(4) $$\begin{array}{lll} \;\mathrm{Pr} & (X,N,A,B,C|\Pi ,V,W)\; & \;\\ & =\mathrm{Pr}(N)\mathrm{Pr}(X,A,B,C|N,\Pi ,V,W)\; & \;\\ & =\mathrm{Pr}(N)\overset{I}{\underset{i=1}{\prod }}\;\;\mathrm{Pr}(X_{i},a_{i},\;B_{i},\;C_{i}|N_{i},\;\Pi ,\;V\;,\;W)\; \end{array}$$

Define $L_{\mathrm{id}0}=\overset{J_{d}}{\underset{j=1}{\prod }}f_{\mathrm{idj}0}(x_{\mathrm{idj}})$, $L_{\mathrm{id}1}=\overset{J_{d}}{\underset{j=1}{\prod }}f_{\mathrm{idj}1}(x_{\mathrm{idj}})$ and $L_{\mathrm{id}2}=\overset{J_{d}}{\underset{j=1}{\prod }}f_{\mathrm{idj}2}(x_{\mathrm{idj}})$. Define *δ*(*.*) to be an indicator function. *δ*(*.*)=1 if its argument is true, and *δ*(*.*)=0 otherwise. We have

(5) $$\begin{array}{lll} & \;\mathrm{Pr}\left(X_{i},a_{i},B_{i},C_{i}|N_{i},\Pi ,V,W\right)\; & \;\\ & \;=\mathrm{Pr}\left(a_{i}|\Pi \right)\;\overset{D}{\underset{d=1}{\prod }}\;\mathrm{Pr}\left(b_{\mathrm{id}},c_{\mathrm{id}}|a_{i},\;V,\;W\right)\;\mathrm{Pr}\;\left(X_{\mathrm{id}}|N_{\mathrm{id}},a_{i},b_{\mathrm{id}},c_{\mathrm{id}}\right)\; & \;\\ & \;={\left\{\;\Pi_{0}\overset{D}{\underset{d=1}{\prod }}L_{\mathrm{id}0}\right\}}^{\delta \left(a_{i}=0\right)}\overset{K}{\underset{k=1}{\prod }}\left\{\Pi_{k}\overset{D}{\underset{d=1}{\prod }}{\left[v_{\mathrm{kd}}L_{\mathrm{id}1}\right]}^{b_{\mathrm{id}}}\;{\left[w_{\mathrm{kd}}L_{\mathrm{id}2}\right]}^{c_{\mathrm{id}}}\right)\; & \;\\ & \;\times {\left({\left[(1-v_{\mathrm{kd}}-w_{\mathrm{kd}})L_{\mathrm{id}0}\right]}^{1-b_{\mathrm{id}}-c_{\mathrm{id}}}\right\}}^{\delta \left(a_{i}=k\right)}\; \end{array}$$

To infer ***Π***, ***V*** and ***W***, we employ a Bayesian approach by imposing a Dirichlet prior *D*(*η*,⋯,*η*) on ***Π*** and imposing independent Dirichlet priors *D*(*η*,*η*,*η*) on all triplets (*v*_*kd*_,*w*_*kd*_,1−*v*_*kd*_−*w*_*kd*_). The joint posterior distribution of unknown parameters and indicators given the observed data is:

(6) $$\begin{array}{lll} \;\mathrm{Pr} & \left(A,\;B,\;C,\;\Pi ,\;V,\;W|X,N\right)\; & \;\\ & \propto \mathrm{Pr}\left(X,N,A,B,C|\Pi ,V,W\right)\;f\left(\Pi ,V,W\right)\; & \;\\ & \propto \overset{I}{\underset{i=1}{\prod }}\;\mathrm{Pr}\;\left(X_{i},a_{i},B_{i},\;C_{i}|N_{i},\;\Pi ,\;V,\;W\right)\left\{\overset{K}{\underset{k=0}{\prod }}\Pi_{k}^{\eta -1}\right\}\; & \;\\ & \;\times \left\{\overset{K}{\underset{k=1}{\prod }}\overset{D}{\underset{d=1}{\prod }}v_{\mathrm{kd}}^{\eta -1}\;w_{\mathrm{kd}}^{\eta -1}{\left(1-v_{\mathrm{kd}}-w_{\mathrm{kd}}\right)}^{\eta -1}\right\}\; \end{array}$$

Conditional on the observed data, *Pr*(***N***) is a constant that does not contain parameters of interest, therefore it is absorbed into a proportionality constant not shown in the formula above. Using this joint posterior, an EM algorithm can be derived to search for posterior mode $\left(\hat{\Pi },\hat{V},\hat{W}\right)$ of $\mathrm{Pr}(\Pi ,V,W|X,N)=\underset{A,B,C}{\sum }\mathrm{Pr}(A,B,C,\Pi ,V,W|X,N)$ in which the missing indictors ***A***, ***B*** and ***C*** are all integrated out (see Additional file 1: Supplemental Method S.4).

For the Dirichlet prior, we use *η*=2 (see Additional file 1: Supplemental Method S.3 for a discussion on the choice of parameter for the Dirichlet prior). In the EM algorithm, we assume that the class number *K* is given. In order to choose the optimal *K*, we run the algorithm multiple times using different values of *K*. We choose the best *K* using the Bayesian Information Criterion (BIC) (see Additional file 1: Supplemental Method S.5).

### Statistical inference of allele-specificity

The estimated ***Π***, ***V*** and ***W*** can describe the correlation patterns of allele-specificity among datasets. Given ***Π***, ***V*** and ***W***, one can infer whether SNP *i* belongs to class *k* based on the posterior probability *Pr*(*a*_*i*_ = *k*|***X***_*i*_,***N***_*i*_,***Π***,***V***,***W***) (see Additional file 1: Supplemental Method S.4 equations S.12-S.13). One can then infer whether each SNP *i* is skewed in each individual dataset *d* based on the posterior probability $\mathrm{Pr}(b_{\mathrm{id}},c_{\mathrm{id}}|X_{i},N_{i},\Pi ,V,W)=\underset{a_{i}}{\sum }\mathrm{Pr}(a_{i},b_{\mathrm{id}},c_{\mathrm{id}}|X_{i},N_{i},$***Π***,***V***,***W***) after summing over all possible values of *a*_*i*_(see Additional file 1: Supplemental Method S.4 equation S.14). Note that

(7) $$\;\begin{array}{l} \mathrm{Pr}\left(b_{\mathrm{id}},\;c_{\mathrm{id}}|X_{i},\;N_{i},\;\Pi ,\;V,\;W\right)=\\ \underset{k}{\sum }\;\mathrm{Pr}\left(a_{i}\;=\;k|X_{i},\;N_{i},\;\Pi ,\;V,\;W\right)\mathrm{Pr}\left(b_{\mathrm{id}},c_{\mathrm{id}}|a_{i}\;=\;k,X_{i},N_{i},\Pi ,V,W\right) \end{array}$$

Define

(8) $$\begin{array}{l} {\tilde{P}}_{\mathrm{id}}=\max\left\{\left(\mathrm{Pr}\left(b_{\mathrm{id}}=1,c_{\mathrm{id}}=0|X_{i},N_{i},\Pi ,V,W\right),\right)\right)\\ \;\left(\left(\mathrm{Pr}\left(b_{\mathrm{id}}=0,c_{\mathrm{id}}=1|X_{i},N_{i},\Pi ,V,W\right)\right)\right\} \end{array}$$

Using ${\tilde{P}}_{\mathrm{id}}$, SNPs can be rank ordered for biologists to choose candidates to design follow-up studies. For each top ranked SNP, one can determine its skewing direction by comparing *Pr*(*b*_*id*_=1,*c*_*id*_=0|***X***_*i*_,***N***_*i*_,***Π***,***V***,***W***) and *Pr*(*b*_*id*_=0,*c*_*id*_=1|***X***_*i*_,***N***_*i*_,***Π***,***V***,***W***). The one with the larger value determines the direction. Finally, the posterior probabilities of top *N* SNPs can be converted to an estimate of false discovery rate (FDR) using $\mathrm{FDR}(N)=\underset{i\in \mathrm{top}\;N\;\mathrm{SNPs}}{\sum }(1-{\tilde{P}}_{\mathrm{id}})/N$.

Formula 7 shows that two types of information contribute to *Pr*(*b*_*id*_,*c*_*id*_|***X***_*i*_,***N***_*i*_,***Π***,***V***,***W***): (1) *Pr*(*a*_*i*_=*k*|***X***_*i*_,***N***_*i*_,***Π***,***V***,***W***), which is determined using information from all *D* datasets, and (2) *Pr*(*b*_*id*_,*c*_*id*_|*a*_*i*_=*k*,***X***_*i*_,***N***_*i*_,***Π***,***V***,***W***), which only uses information specific to dataset *d* conditional on ***Π***, ***V*** and ***W***. Thus for each particular dataset *d*, the dataset-specific information is weighted by information obtained from other datasets to determine the SNP ranking. Intuitively, if allelic imbalance in two datasets are correlated, then observing an AS event in one dataset will suggest that a relatively weak skewing event observed at the same SNP in the other dataset is very likely to be a true AS event. In contrast, if no AS event is observed in one dataset, then a relatively weak skewing event observed at the same SNP in the other dataset is likely to be a false positive. This is the underlying nature of using *Pr*(*a*_*i*_=*k*|***X***_*i*_,***N***_*i*_,***Π***,***V***,***W***) to re-weigh information in *Pr*(*b*_*id*_,*c*_*id*_|*a*_*i*_=*k*,***X***_*i*_,***N***_*i*_,***Π***,***V***,***W***), and it provides the foundation for improving SNP ranking by borrowing information across datasets. In real applications, ***Π***, ***V***,***W*** are unknown, and they are replaced by the posterior mode obtained from the EM algorithm.

## Results

### GM12878 data and preprocessing

We collected 40 ENCODE [36] ChIP-seq datasets with a total of 77 samples together with a genomic DNA sample in GM12878 lymphoblastoid cells (Additional file 2: Table S1). GM12878 is a female and is one of the most extensively studied cell lines in ENCODE. Within each dataset, the number of replicate samples varied from 1 to 3. We downloaded the raw sequence reads of all 78 samples and mapped them to human genome (hg18) (see details in Additional file 1). We removed repeated sequences from the ChIP-seq datasets to avoid PCR duplicates, which may skew the determination of allelic biases. In other words, if multiple reads have exactly the same sequence, only one copy is retained. We obtained the genotype data for GM12878 from [37].

As previously described in [31], there are two different types of read mapping biases that may affect the analysis of AS events: the reference bias and the inherent bias. The reference bias often occurs when one maps sequence reads to a reference genome. If one allows the same number of mismatches in the alignment, a read from the non-reference allele is less likely to be mapped back to the reference genome compared to a read from the reference allele, since the non-reference read has one more mismatch to the reference genome. This phenomenon is known as the reference bias. This type of bias, if it exists, is automatically taken care of by the iASeq model through the parameter *p*_*dj*0_ which models the background skewing probability and is estimated using all reads mapped to heterozygote SNPs in each sample. If there is reference mapping bias, *p*_*dj*0_will take a value different from 0.5 to adjust for the bias. One may remove reference bias before the analysis by masking SNPs in the reference genome during the alignment or by aligning reads to a diploid personal genome. This situation will also be automatically recognized by iASeq through the estimation of *p*_*dj*0_ from the data (if there is no bias, *p*_*dj*0_=0*.*5). Therefore, regardless of whether the reference bias has been removed from the data in the preprocessing or not, the iASeq model is able to automatically handle it and adjust the inference accordingly.

The intrinsic bias is a different type of bias. As shown by [31], even if the reference bias is removed (e.g., by masking SNPs in the reference genome), the inherent bias still exists. For example, suppose sequence 1 (e.g., xxxAxxx) and sequence 2(e.g., xxxTxxx) are two reads that differ only in one position (i.e., A/T). It is possible that sequence 1 is easier to be mapped back to its correct location in the genome than sequence 2 if the second sequence has many repeats in the genome. This bias reflects the inherent characteristics of the genome and cannot be removed by masking variants in the reference genome or by mapping reads to a diploid personal genome. In the above example, masking A and T in the original reads is also not a solution, since a priori one does not know which position in a read corresponds to a SNP position and therefore should be masked without first aligning the read to the genome. When a heterozygote SNP has inherent bias, one allele will have higher read counts than the other even if the two alleles have the same binding level. To avoid this bias, we used the approach described in [22,31] to remove SNPs with the inherent bias.

We began with 1,704,166 heterozygote SNPs and filtered out 149,996 (8.8%) SNPs with inherent bias. Next, we eliminated SNPs that were not bound by any TF or associated with any HM in any dataset (see Additional file 1: Supplemental Methods S.1.1, S.1.2 and Additional file 3: Table S2 for details). After applying these filters, 94,519 heterozygote SNPs remained. These 94,519 SNPs were then analyzed by iASeq.

### A simulation study

**Figure 2:**
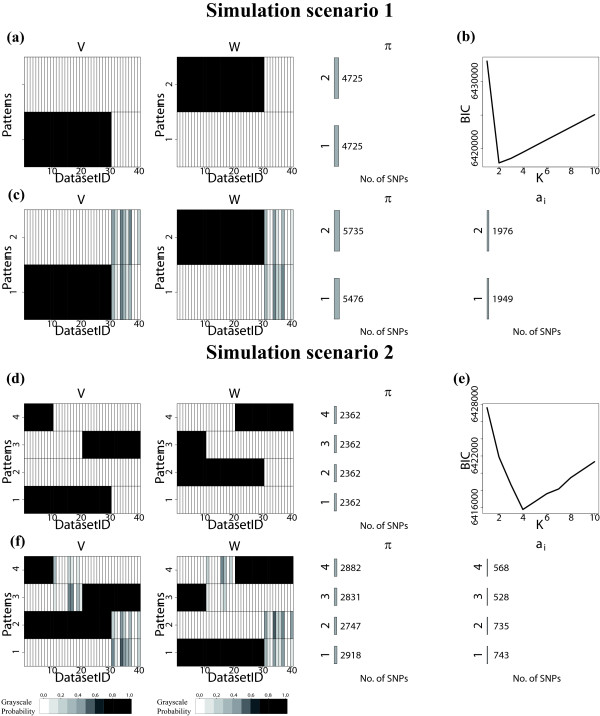
**Simulation design and patterns discovered by iASeq. (a)** The true ASB patterns in simulation 1. Two patterns were simulated in addition to the background pattern. The two non-background patterns are shown. Each pattern has 4725 SNPs. Each row in the plot represents a SNP class, and each column represents a dataset. Black means skewed, and white means not skewed. **(b)** The BIC values for different class number *K* in simulation 1. The BIC achieves the minimum at *K*=2. **(c)** Patterns discovered by iASeq in simulation 1. The plot shows the estimated ***V*** and ***W*** when *K*=2. Each row corresponds to a class. Each column represents a dataset. The color in the cell (*k*,*d*) demonstrates the estimated SR or SN probability in class *k* and dataset *d*. From white to dark, the probability increases from 0 to 1. The numbers shown under *Π*are the estimated number of SNPs in each class (i.e., ${\hat{\Pi }}_{k}*$ the total number of SNPs). The numbers shown under *a*_*i*_are the number of SNPs identified for the corresponding class using the posterior probability *Pr*(*a*_*i*_=*k*|***X***_*i*_,***N***_*i*_,***Π***,***V***,***W***)>0*.*9 as cutoff. **(d)** The true ASB patterns in simulation 2. Four patterns were simulated in addition to the background pattern. The four non-background patterns are shown. Each pattern has 2362 SNPs. **(e)** The BIC values for different class number *K* in simulation 2. The BIC achieves the minimum at *K*=4. **(f)** The patterns discovered by iASeq in simulation 2.

Before we apply iASeq to the real data, we first tested its performance in simulations that took into account real data characteristics. Our simulations kept the same design as the real GM12878 ChIP-seq data, with the same number of datasets and the same number of replicates within each dataset, except that the genomic DNA sample was not used here since we knew the truth in the simulations and did not need genomic DNA as a control for potential bias. To create the simulation data, we first applied iASeq to the real GM12878 data to identify 86,353 SNPs that were not skewed in any dataset using *Pr*(*a*_*i*_=0|***X***_*i*_,***N***_*i*_,***Π***,***V***,***W***)>0*.*5 as cutoff. To mimic the real background noise, these SNPs were resampled by a bootstrap procedure to create the background SNPs in the simulations, and we kept the read counts (*x*_*idj*_,*n*_*idj*_) of each background SNP as is in the simulated data. Next, we simulated ASB SNPs and added them to the background. Simulations were carried out under two different scenarios (Figure 2). 

● Scenario 1: Two types of ASB SNPs (classes 1 and 2) were created in addition to the background SNPs (class 0). The SNP number for class 0, 1, and 2 was 85,069, 4,725 and 4,725 respectively. Thus the true *Π*_*k*_for the three classes was 0.90, 0.05 and 0.05 respectively. SNPs in class 1 were SR in datasets 1 to 30 (i.e., their *b*_*id*_=1 for *d*=1,⋯,30). SNPs in class 2 were SN in datasets 1 to 30 (i.e., *c*_*id*_=1 for *d*=1,⋯,30). In datasets 31 to 40, no SNPs had allelic imbalance. Class 2 can be viewed as the mirror image of class 1. This symmetric design reflects the symmetry of allele-specificity, that is, the skewing to the reference allele and to the non-reference allele is approximately symmetric. The class abundance (0.90,0.05,0.05) roughly matched the abundance observed in the analysis of real GM12878 data.

● Scenario 2: Four correlation patterns (classes 1-4) were created in addition to the background class (class 0). Class 1 and class 2 were the same as in simulation 1. Classes 3 and 4 were two new patterns. SNPs in class 3 were SR in datasets 21-40, and SN in datasets 1-10. Class 4 was the mirror image of class 3. The abundance of the classes 0 to 4 was (0.90,0.025,0.025,0.025,0.025).

Given the simulated [***B***_*i*_;***C***_*i*_] configurations, we then simulated the read count data for ASB SNPs as described in detail in Additional file 1: Supplemental Methods S.6. Simulations done in this way was able to keep the major characteristics of real data while allowing us to benchmark the performance of different methods since we knew the truth.

**Figure 3:**
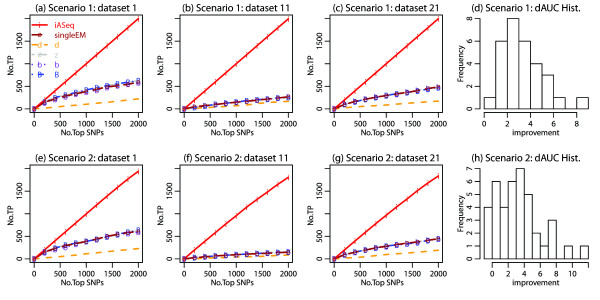
**The Receiver Operating Characteristic (ROC) curves for simulations. (a)-(c)** We plot the number of true allele-specific SNPs (i.e., true positives, TP) among the top *q* ranked SNPs in each dataset against the rank cutoff *q*. Results for different methods in three representative datasets in simulation 1 are shown. Results in all other datasets were similar. **(d)** For each ranking method and each dataset, we computed the area under the ROC curve (AUC) using the 2000 top ranked SNPs. dAUC, the proportion of improvement of AUC brought by iASeq over the best AUC obtained from the single-dataset based methods, was computed for each dataset. *dAUC*>0 means iASeq brings improvement. The distribution of dAUC in all 40 datasets is shown for simulation 1. **(e)**-**(g)** Results in three representative datasets from simulation 2. Results in all other datasets were similar. **(h)** The distribution of dAUC in all 40 datasets is shown for simulation 2.

We applied iASeq to both simulations. In both cases, iASeq was able to identify the correct number of SNP classes using BIC (Figures 2a,b,d,e). Figures 2c and 2f show that the ASB patterns reported by iASeq matched the true patterns well. In order to test whether iASeq can improve the statistical power of detecting SNPs with allelic imbalance, we compared the SNP ranking provided by iASeq with rankings provided by five other methods that analyze each dataset separately (Figure 3). In iASeq, SNPs were ranked in each dataset *d* according to the posterior probability ${\tilde{P}}_{\mathrm{id}}$ defined by Formula 8. Since we know the truth, we can count how many of the top *N* SNPs were true positives. Here the true positives were defined as SNPs that were truly allele-specific and also had the skewing direction correctly inferred. The five single-dataset based methods for ranking SNPs include a *deviation statistic d*, *naive z statistic*, *naive Bayes statistic*, *empirical Bayes statistic* and *single dataset EM*. These methods were applied to each individual dataset. For each dataset *d*, we merged data from all replicates to obtain $x_{\mathrm{id}}=\overset{J_{d}}{\underset{j=1}{\sum }}x_{\mathrm{idj}}$ and $n_{\mathrm{id}}=\overset{J_{d}}{\underset{j=1}{\sum }}n_{\mathrm{idj}}$. We then computed the statistics used for SNP ranking as described below. 

1. *Deviation statistic (d)*: SNPs were ranked based on |*x*_*id*_/*n*_*id*_−*p*_*d*0_|. Here we estimated $p_{d0}=\frac{1}{I^{\prime }}\underset{i:n_{\mathrm{id}}\neq 0}{\sum }p_{\mathrm{id}}=\frac{1}{I^{\prime }}\underset{i:n_{\mathrm{id}}\neq 0}{\sum }\frac{x_{\mathrm{id}}}{n_{\mathrm{id}}}$, where *I*^*′*^is the number of SNPs for which *n*_*id*_≠0.

2. *Naive z statistic (z)*: SNPs were ranked based on $\frac{\left|x_{\mathrm{id}}/n_{\mathrm{id}}-p_{d0}\right|}{\sqrt{\left(p_{d0}*(1-p_{d0})/n_{\mathrm{id}}\right)}}$. Here *p*_*d*0_was estimated as in the *deviation statistic d*.

3. *Naive Bayes statistic (b)*: SNPs were ranked using $\left|(x_{\mathrm{id}}+2*{\tilde{p}}_{d0})/(n_{\mathrm{id}}+2)-{\tilde{p}}_{d0}\right|$. Here ${\tilde{p}}_{d0}=\frac{1}{I}\underset{i}{\sum }\frac{x_{\mathrm{id}}+2*p_{d0}}{n_{\mathrm{id}}+2}$ where *p*_*d*0_was estimated as in the *deviation statistic d*. The implicit assumption here is that *x*_*id*_|*p*_*id*_∼*Bin*(*n*_*id*_,*p*_*id*_) and *p*_*id*_∼*Beta*(*α*_*d*_,*β*_*d*_) with $\alpha_{d}=2{\tilde{p}}_{d0}$ and $\beta_{d}=2(1-{\tilde{p}}_{d0})$. The posterior mean of *p*_*id*_is used to construct the ranking statistic.

4. *Empirical Bayes statistic (B)*: SNPs were ranked using $\left|(x_{\mathrm{id}}+{\hat{\alpha }}_{d})/(n_{\mathrm{id}}+{\hat{\alpha }}_{d}+{\hat{\beta }}_{d})-{\overset{ˇ}{p}}_{d0}\right|$. We estimated ${\overset{ˇ}{p}}_{d0}=\frac{{\hat{\alpha }}_{d}}{{\hat{\alpha }}_{d}+{\hat{\beta }}_{d}}$. The implicit assumption is the same as the *naive Bayes statistic*, but now we estimate *α*_*d*_and *β*_*d*_based on the observed data using the method of moments as in iASeq (see Additional file 1: Supplemental Method S.2).

5. *Single dataset EM (singleEM)*: We fitted a mixture model of SR, SN and NS with distributions *f*_*idjp*_(·),*p*=0,1,2 and mixing probabilities *v*_*d*_, *w*_*d*_and 1−*v*_*d*_−*w*_*d*_for each dataset *d* without considering other datasets. SNPs were ranked using a posterior probability similar to ${\tilde{P}}_{\mathrm{id}}$, but now determined based on information in dataset *d* only (see Additional file 1: Supplemental Method S.7 for details).

Figure 3 compares the number of true positives, *TP*_*d*_(*q*), in the top *q* SNPs reported by each method in each dataset *d*. In Figures 3a-c and 3e-g, *TP*_*d*_(*q*) is plotted as a function of *q* in a few representative datasets. These plots show that iASeq outperformed all single-dataset based methods, and it was able to substantially improve the power for detecting allele-specificity.

In general, the observed differences between iASeq and the *d, z, b* and *B* statistics could be caused by many factors such as use of different statistical models, ranking statistics, or methods for parameter estimation. However, the comparison between iASeq and the single dataset EM represents a well-controlled comparison since these two methods used exactly the same distributional assumptions and parameter estimation methods. The only difference between them was that iASeq used information from multiple datasets whereas *singleEM* was based on one dataset only. This well-controlled comparison shows that jointly modeling multiple datasets is able to improve the allelic inference.

To examine whether iASeq was able to bring improvement in all datasets, we computed the Area under the Receiver Operating Characteristic (ROC) curves (AUC) for each method in each dataset using the top 2000 ranked SNPs. In each dataset, we computed the proportion of improvement in terms of AUC brought by iASeq over the best single-dataset based ranking method (i.e., $\mathrm{dAUC}=\frac{\mathrm{AU}C_{\text{iAseq}}-\mathrm{AU}C_{\text{bestsingle}}}{\mathrm{AU}C_{\text{bestsingle}}}$). *dAUC*>0 means iASeq is able to bring improvement. Figures 3d and 3h show the distribution of *dAUC* across all 40 datasets as a histogram. The results show that iASeq was able to improve the SNP ranking in almost all datasets.

**Figure 4:**
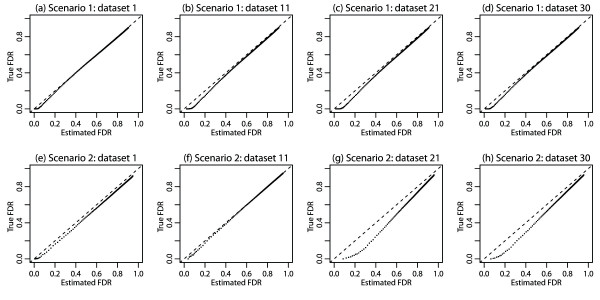
**Estimated FDR against true FDR in simulations. (a)-(d)** Results for four representative datasets in simulation 1. **(e)**-**(h)** Results for four representative datasets in simulation 2. Results for all other datasets were similar.

In Figure 4, we converted the iASeq posterior probabilities of top *N* SNPs to FDR estimates and plotted the estimated FDR against the true FDR. The figure shows that iASeq was able to provide reasonable FDR estimates as well. Shown in the figure are a few representative datasets. Results in all other datasets were similar.

### Analysis of real data

Our simulation study demonstrates the ability of iASeq to discover correlation patterns of allele-specificity and improve the detection of skewed SNPs. Next, we applied iASeq to analyze the 41 real datasets (78 samples) in GM12878 cells. In real data, we do not have comprehensive knowledge about the truth. Therefore, unlike simulations, we were not able to assess the FDR estimates. For this reason, we mainly focused on analyzing the correlation patterns of allele-specificity and testing whether iASeq can improve the SNP ranking.

#### Correlation patterns of allele-specificity

**Figure 5:**
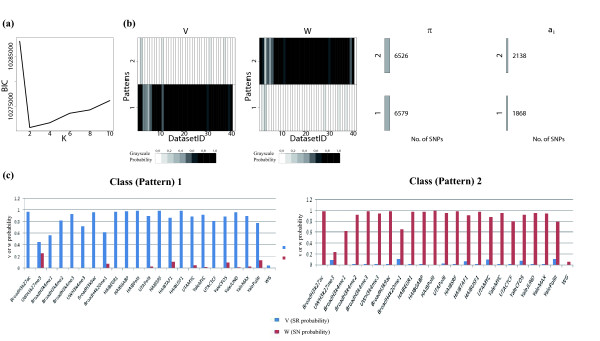
**Correlation patterns of allele-specificity among different TFs and HMs in GM12878 cells discovered by iASeq. (a)** The BIC values for different class number *K*. The BIC achieves the minimum at *K*=2. **(b)** The estimated ***V*** and ***W*** when *K*=2. Each row corresponds to a class. Each column represents a dataset. The color in the cell (*k*,*d*) represents the SR or SN probability in class *k* and dataset *d*. From white to dark, the probability increases from 0 to 1. The bar plot and the numbers shown under *Π*are the estimated number of SNPs in each class (i.e., ${\hat{\Pi }}_{k}*$ the total number of SNPs). The bar plot and the numbers shown under *a*_*i*_are the number of SNPs identified for the corresponding class using the posterior probability *Pr*(*a*_*i*_=*k*|***X***_*i*_,***N***_*i*_,***Π***,***V***,***W***)>0*.*9 as cutoff. **(c)** A closer look at ***V*** and ***W*** in a number of representative datasets. The barplots show the estimated SR and SN probabilities *v*_*kd*_and *w*_*kd*_in a number of selected datasets. Left: the skewing probabilities in class 1. Right: the skewing probabilities in class 2. The height of each bar represents the SR or SN probability.

Figure 5a shows the BIC in the real data. Based on BIC, the optimal *K* was 2. In other words, in addition to the background class (*k*=0), iASeq discovered two other SNP classes, representing different allele-specificity patterns. For these two non-background classes, *Π*_*k*_was estimated to be 0.0696 and 0.0691 respectively, suggesting that they cover 6.96% and 6.91% of the analyzed SNPs. Due to the background noises, not all SNPs in these two classes can be confidently detected. At the 0.90 posterior probability cutoff, iASeq reported 1868 and 2138 SNPs for classes 1 and 2 respectively (Figure 5b). Note that our simulations had similar settings as the real data analysis, and they showed that iASeq was able to discover more than two patterns if they are supported by the data. Therefore our discovery of two correlation patterns here is likely driven by the data, that is, the information in the data is only sufficient for supporting robust discovery of two patterns.

Figures 5b and 5c show the posterior mode of ***V***_*k*_and ***W***_*k*_ for the two non-background classes. It turned out that these two classes corresponded to two global directions of allele-specificity, SR and SN, respectively. Since the assignment of reference or non-reference allele depends on the reference genome, the assignment *per se* is not of biological interest. However, recall that GM12878 is a single person, therefore at each single SNP, the nucleotide representing the reference or non-reference allele is the same across all datasets analyzed here. Given this fact, what these results essentially tell is that at each single SNP, most TFs and HMs in our analysis were highly correlated in terms of allele-specificity, and if they are skewed, they tend to be skewed toward the same direction (i.e., the same allele). For instance, for SNPs in class 1, both H3K4me3 (from the Broad Institute) and H3K27ac (Broad) had high probability to be SR, with (*v*_*kd*_,*w*_*kd*_) equal to (0.9337,0.0070) and (0.9730,0.0041) respectively (Figure 5c). The probability that one is SR and the other one is SN was small. Similarly, for SNPs in class 2, both H3K4me3 and H3K27ac were highly likely to be SN simultaneously ((*v*_*kd*_,*w*_*kd*_)=(0.0061,0.9835) for H3K4me3 (Broad) and (0.0040,0.9897) for H3K27ac (Broad)). While the allelic imbalance of most TFs and HMs were highly correlated, H3K27me3, a HM involved in gene repression, was an exception. In both non-background classes, H3K27me3 had much lower skewing probabilities compared to the other proteins (Figure 5c). Within each class, the difference in the skewing probability between the two alleles was also much weaker for H3K27me3 as compared to the other proteins. For instance, in class 1, while most other proteins showed strong preference to be skewed toward the reference allele, H3K27me3 can be skewed to the reference allele at some SNPs and skewed to the non-reference allele at many other SNPs. Therefore, the allelic imbalance in H3K27me3 is not strongly correlated with the allelic imbalance of the other proteins analyzed here. For the genomic DNA which was used as control here, the skewing probabilities (*v*_*kd*_,*w*_*kd*_) in both classes were fairly low as shown in Figure 5b-c. In both classes, the probability for not being skewed in the genomic DNA (i.e., 1−*v*_*kd*_−*w*_*kd*_) was bigger than 0.95. This indicates that the high probability of skewing observed in the other datasets was not an artifact.

The coordinated allelic imbalance of different proteins toward the same allele has also been observed in a recent study [38]. In that study, the authors analyzed AS of 24 TFs and found that when multiple TFs bind to the same SNP, they frequently bind to the same allele. Moreover, those authors did not observe any pair of TFs that regularly bind the same position on alternate alleles. Our observation here therefore is consistent with their finding.

#### Increased power for detecting allele-specificity compared with single dataset analysis

We ranked SNPs based on the posterior probabilities ${\tilde{P}}_{\mathrm{id}}$ in each dataset. The iASeq ranking was compared with the rankings provided by the five single-dataset based methods described above. Since we do not know the truth, we used two types of independent information as gold standard to benchmark the ranking results.

**Figure 6:**
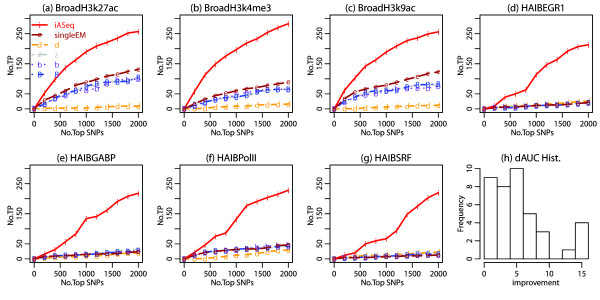
**The ROC curves with chrX-npa SNPs as gold standard in the GM12878 analysis.** We plot the number of non-pseudoautosomal region X chromosome SNPs, denoted by *TP*_*d*_(*q*), among the top *q* ranked SNPs in dataset *d* as a function of the rank cutoff *q* for each method. **(a)**-**(g)** Results in 7 representative datasets. **(h)** In each dataset, we computed the area under the ROC curve (AUC) using the 2000 top ranked SNPs for each method. dAUC, the proportion of improvement of AUC brought by iASeq over the best AUC from the single-dataset based methods, was computed for each dataset. The distribution of dAUC in all 40 datasets is shown.

First, we evaluated different methods by counting how many of their top ranked SNPs were located in the non-pseudoautosomal regions of chromosome X (chrX-npa) (Figure 6). GM12878 is a female lymphoblastoid cell line. In GM12878, SNPs in chrX-npa are expected to be allele-specific due to cells rapidly become clonal in culture leading to a skewed X-inactivation [5,38,39]. Therefore, given a fixed number of top SNPs, the more chrX-npa SNPs one can find, the more powerful a method is. Figure 6 shows that iASeq clearly increased the power for detecting allele-specificity in each dataset compared to the single-dataset based analysis. For example, Figure 6a shows that in the H3K27ac dataset generated by the Broad Institute, iASeq was able to identify 122 chrX-npa SNPs among the top 500 SNPs. This represents 126% improvement compared to *singleEM*, the best single-dataset based ranking method in that dataset, which only identified 54 chrX-npa SNPs. Figures 6a-g show results in a few representative datasets. Figure 6h shows the distribution of *dAUC* (i.e., the proportion of improvement of AUC by iASeq over the best single-dataset based ranking method in each dataset) in all 40 datasets. These plots clearly show that iASeq outperformed all single-dataset based methods in all datasets and the average improvement in AUC was 354%.

**Figure 7:**
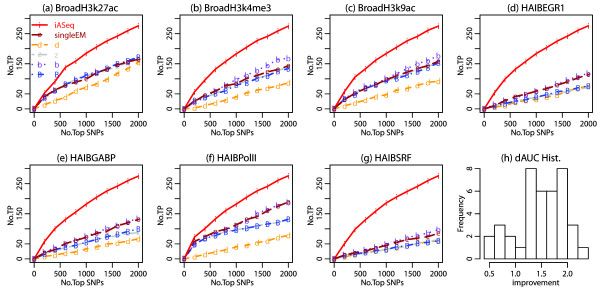
**The ROC curves in GM12878 data using Caltech RNA-seq ASE SNPs as gold standard.** We plot *TP*_*d*_(*q*), the number of true allele-specific SNPs among the top *q* ranked SNPs in dataset *d*, against the rank cutoff *q* for each method. The true allele-specific SNPs are defined as SNPs that have ≥1 RNA-seq exonic ASE SNPs in their 10kb neighborhood. **(a)**-**(g)** Results in 7 representative datasets. **(h)** In each dataset, we computed the area under the ROC curve (AUC) using the 2000 top ranked SNPs for each method. dAUC, the proportion of improvement of AUC brought by iASeq over the best AUC from the single-dataset based methods, was computed for each dataset. The distribution of dAUC in all 40 datasets is shown.

Second, we evaluated different methods by using independent RNA-seq data. From RNA-seq, one can identify exonic ASE SNPs and use them as gold standard. We collected two RNA-seq datasets in GM12878, one from the California Institute of Technology (Caltech) and the other from the Yale/Stanford University (Yale) (Additional file 2: Table S1). From each dataset, we identified the top 400 exonic ASE SNPs using the naive Bayes statistics. Using the other methods to identify the gold standard ASE SNPs produced similar results which, for simplicity, will not be shown here. Based on these exonic ASE SNPs, we defined a SNP in our ChIP-seq analysis as truly allele-specific if there was an exonic ASE SNP in its *X*kb neighborhood. Here we tried both *X*=10kb and *X*=1kb and obtained similar results. Below we illustrate the results using *X*=10kb as an example. The corresponding results for *X*=1kb are shown in Additional file 4: Figures S3-S6. Among the 94,519 SNPs analyzed in the ChIP-seq data, 20,526 had one or more exonic SNPs within its 10kb neighborhood and therefore could potentially be linked to an exonic ASE SNP. Figure 7 and Additional file 5: Figure S1 compare rankings of these SNPs provided by different methods in terms of how many of the top ranked SNPs are true positives (i.e., associated with ASE). iASeq again outperformed all the other single-dataset based ranking methods. For instance, based on the Caltech gold standard, iASeq on average identified 144% more true positive SNPs among the top 500 SNPs (Figure 7a-g). According to the Yale gold standard, iASeq achieved an average of 149% improvement in terms of the true positive rate among top 500 SNPs (Additional file 5: Figure S1). The average improvement in terms of AUC (i.e., *dAUC*) across all 40 datasets was 148% (Figure 7h) and 165% (Additional file 5: Figure S1h) for the Caltech and Yale gold standard respectively.

**Figure 8:**
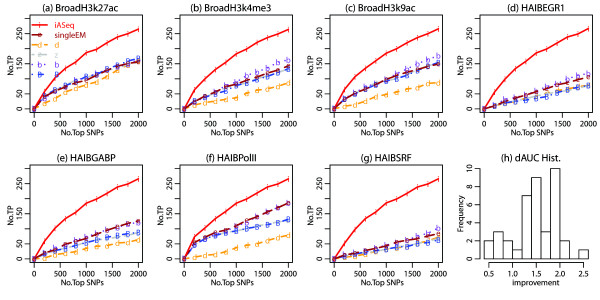
**The ROC curves in GM12878 data using Caltech RNA-seq autosomal ASE SNPs as gold standard.** We plot *TP*_*d*_(*q*), the number of true allele-specific SNPs among the top *q* ranked autosomal SNPs in dataset *d*, against the rank cutoff *q* for each method. The true allele-specific SNPs are defined as autosomal SNPs that have ≥1 RNA-seq exonic ASE SNPs in their 10kb neighborhood. **(a)**-**(g)** Results in 7 representative datasets. **(h)** In each dataset, we computed the area under the ROC curve (AUC) using the 2000 top ranked SNPs for each method. dAUC, the proportion of improvement of AUC brought by iASeq over the best AUC from the single-dataset based methods, was computed for each dataset. The distribution of dAUC in all 40 datasets is shown.

To ensure that the increased statistical power was not completely attributed to X chromosome SNPs, we repeated the benchmark analysis based on RNA-seq using only SNPs in autosomal chromosomes, and we obtained similar results (Figure 8, Additional file 6: Figure S2). This shows that the increased power is not only contributed by chrX SNPs.

#### Comparisons with other methods

Most existing studies on allele-specificity were conducted using in-house data analysis pipelines. A tool developed by Skelly et al. [33] and AlleleSeq [32] are two software tools accessible to third-party users for AS analysis. The method proposed by Skelly et al. [33] is designed for analyzing ASE in RNA-seq data. It first fits a background model using genomic DNA and then feeds the estimated parameters into a Bayesian model that combines information from multiple SNPs within a gene to infer ASE. When we applied this method to analyzing the GM12878 ChIP-seq data, two problems occurred. First, the method uses Markov Chain Monte Carlo (MCMC) to fit the background model from the genomic DNA which, as alerted by [33], is well-known for its slow speed and difficulties for users to monitor the convergence. Our genomic DNA data had 94,519 SNPs which covered 12,417 genes. Running this algorithm on this data using the parameter settings recommended by [33] on a machine with 2.7 GHz CPU and 4 Gb RAM took more than 60 days. Second, after feeding the background model parameters obtained from the first step to the inference model in the second step, the algorithm stopped execution after a few iterations. This is because the original model was developed for deeply sequenced RNA-seq rather than ChIP-seq, where the average read count covering a heterozygote SNP in a ChIP-seq dataset is only 0.64. As a result, the model developed in [33] did not fit the real data in ChIP-seq experiments. This lack-of-fit caused the program to stop early, likely due to the abnormally fitted parameters causing various computation problems (e.g., overflow). For this reason, although the method proposed by [33] represents an advanced solution for analyzing RNA-seq ASE, it cannot be directly used to analyze ASB in ChIP-seq data without significantly redesigning the model and algorithm. For this reason, it is not further compared here.

AlleleSeq [32] is another tool for AS analysis. It has been used to analyze ASB of several TFs in GM12878 [32]. AlleleSeq is more focused on the preprocessing step. Its pipeline first constructs a diploid personal genome sequence according to family trio data and then maps ChIP-seq reads to this personal genome. After removing various biases, the method then analyzes allele-specificity in each individual ChIP-seq dataset separately. [32] applied AlleleSeq to analyze 7 different TF datasets in GM12878, among them 5 were also included in our iASeq analysis. We compared iASeq and AlleleSeq using these same 5 datasets. We first obtained the ASB SNPs reported by AlleleSeq from [32]. Let *T*_*d*_denote the number of reported ASB SNPs for each TF dataset *d*. We next obtained the top *T*_*d*_SNPs ranked by iASeq. We then compared these two methods based on how many of their top *T*_*d*_ SNPs were in chrX-npa, and how many of them were associated with exonic ASE SNPs determined by RNA-seq. For the benchmark analysis based on RNA-seq, we associated exonic ASE SNPs with ChIP-seq SNPs using both 10kb and 1kb neighborhood. We also performed the comparison after excluding the chromosome X SNPs. Table 1, Additional file 7: Table S3, Additional file 4: Tables S4-S5 and Additional file 8: Figure S7 show that iASeq either outperformed or performed comparable to AlleleSeq in all datasets. Sometimes, the improvement was substantial (e.g., YaleMYC in Table 1).

**Table 1:** Comparison of iASeq and AlleleSeq

**Gold standard**	**ChrX**			**All Caltech ASE exonic SNPs**			**Autosomal Caltech ASE exonic SNPs**		
**TF**	**T**_**d**_	**AlleleSeq**	**iASeq**	**T**_**d**_	**AlleleSeq**	**iASeq**	**T**_**d**_	**AlleleSeq**	**iASeq**
YaleCFOS	41	3	4	9	5	3	9	5	3
YaleMYC	122	9	22	39	5	10	38	5	10
YaleJUND	289	13	31	24	4	8	23	4	7
YaleMAX	105	3	18	18	3	1	18	3	2
YalePolIII	25	2	2	0	0	0	0	0	0

Column 1: TF name. Column 2: *T*_*d*_is the number of AlleleSeq reported ASB SNPs. Columns 3-4: the number of non-pseudoautosomal region X chromosome SNPs among the top *T*_*d*_allele-specific SNPs reported by AlleleSeq and iASeq. Column 5: *T*_*d*_is the number of AlleleSeq reported ASB SNPs that had an exonic SNP within their 10kb neighborhood. Columns 6-7 show among the top *T*_*d*_allele-specific SNPs reported by AlleleSeq and iASeq, how many SNPs had ≥1 exonic ASE SNP in their 10kb neighborhood according to the Caltech RNA-seq experiment. Column 8: *T*_*d*_is the number of AlleleSeq reported autosomal ASB SNPs that had an exonic SNP within their 10kb neighborhood. Columns 9-10 show among the top *T*_*d*_autosomal allele-specific SNPs reported by AlleleSeq and iASeq, how many SNPs had ≥1 exonic ASE SNP in their 10kb neighborhood according to the Caltech RNA-seq experiment. Additional file 7: Table S3 contains similar results using Yale RNA-seq exonic ASE SNPs as gold standard.

## Discussion

### Interpretation of the correlation patterns

When analyzing the real data in GM12878, iASeq found two non-background AS patterns, representing two opposite directions of allelic imbalance. Since the assignment of reference and non-reference allele depends on the reference genome, whether a SNP is skewed toward reference or non-reference allele *per se* does not have direct biological meaning. What these two patterns essentially suggest is that the allelic imbalances of multiple proteins at a single SNP are correlated and have high preference to be skewed toward the same allele. In other words, the two patterns should be viewed as a pair and interpreted together.

In general, although one may view different allelic imbalance patterns in iASeq as different clusters of SNPs, these clusters only describe the similarities among SNPs in terms of their skewness directions, rather than the similarities in terms of their functions. The direction is defined using the reference/non-reference allele. The reference or non-reference allele for different SNPs can have different meanings (e.g., for one SNP, the maternal allele may be the reference allele, whereas for another SNP the paternal allele may be the reference allele). Therefore within each cluster, even though SNPs have similar skewness pattern, they are not necessarily functionally related to each other. One should not confuse the SNP clusters here with the clusters obtained from the traditional gene expression microarray data analysis, where co-expressed genes in a cluster often have similar functions. In iASeq, the clusters only serve as a tool to describe the correlation structure among different datasets (i.e., proteins), rather than the functional correlation among different SNPs. The correlation patterns among datasets are used by iASeq to inform one how to integrate information across datasets (i.e., which datasets are highly correlated and therefore can borrow information from each other) to improve detection of AS events for each individual SNP and dataset. In order to understand functions of the detected AS events, one needs to further correlate the iASeq results with other information (e.g., one may determine the parent-of-origin of each SNP first and then study various phenomena such as imprinting).

Our observation that different proteins prefer to be skewed in the same direction is consistent with a recent observation reported in [38] that AS of 24 different TFs are skewed toward the same allele. A number of factors could contribute to the observed correlation. First, biologically it is plausible that functionally related HMs and TFs have correlated allele-specificity. For instance, both H3K4me2 and H3K4me3 are markers for active transcription. Therefore, for a specific SNP, if the reference allele is associated with a gene with active transcription but the non-reference allele is not, then it is very likely that both H3K4me2 and H3K4me3 will be skewed toward the reference allele. For another SNP, if the non-reference allele is transcribed but the reference allele is not, then both H3K4me2 and H3K4me3 will have high probability to be skewed toward the non-reference allele. In the genome, H3K4me2 and H3K4me3 are skewed toward reference allele for some SNPs, and skewed toward non-reference allele for some other SNPs. Therefore the skewed SNPs could naturally fall into two clusters, representing two opposite AS directions. Second, as pointed out by [38], the coordinated AS could also occur as a result of the difference in the chromatin landscape between the two alleles. For instance, if the chromatin on one allele is more open and accessible, it could increase the overall binding probability of multiple different proteins, leading to correlated allelic skewing.

While our results show that most analyzed TFs/HMs tend to be skewed toward the same direction, these results do not imply that these proteins are perfectly correlated in terms of allele-specificity at each and every SNP. In iASeq, the correlation patterns ***V***_*k*_and ***W***_*k*_are probabilistic patterns rather than 0-1 vectors. Each correlation class *k* can generate all 3^*D*^ AS configurations. For instance, for a class with [***V***_*k*_;***W***_*k*_]= [(0.9,0.9,0.9,0.1); (0.1,0.1,0.1,0.1)], it is possible to have one SNP with configuration [*SR*,*SR*,*NS*,*NS*] and at the same time another SNP with configuration [*SR*,*NS*,*SR*,*NS*]. Therefore, SNPs in the same class are not required to have the same AS configuration, even though they tend to have similar AS configurations. The probabilistic patterns are used here to provide a parsimonious description of the complex correlation structure in the data, so that one can circumvent the difficulty of handling 3^*D*^ AS configurations whose complexity increases exponentially. As a consequence of using this parsimonious model, multiple weak correlation patterns without strong enough data support could be merged into a bigger class. For instance, consider two AS patterns [***V***_*k*_;***W***_*k*_]=[(1,1,0,0);(0,0,0,0)] (i.e., [SR,SR,NS,NS]) and [***V***_*k*_;***W***_*k*_]=[(0,0,1,1);(0,0,0,0)] (i.e., [NS,NS,SR,SR]). Suppose both patterns are equally likely to occur in the data. If each pattern is only associated with a small number of SNPs, then a parsimonious model will prefer merging them together into one single class with [***V***_*k*_;***W***_*k*_]=[(0*.*5,0*.*5,0*.*5,0*.*5);(0,0,0,0)]. For this reason, iASeq only discovers correlation patterns that have sufficient data support so that they can be distinguished from other patterns. It will not report weak patterns, which could be real but do not have enough data support to allow them to be robustly recovered. For users, this means that at the cluster level, they may not be able to see weak but real AS correlation patterns if these patterns are not associated with enough number of SNPs. On the other hand, for the purpose of inferring whether or not each SNP is allele-specific in each dataset, these parsimonious correlation patterns are sufficient for describing the correlation structure in the data and serving as a prior to guide the information sharing across datasets. The information sharing will lead the increased ASB detection power, and the eventual AS configuration at each individual SNP will be determined by the posterior probabilities of (*b*_*id*_,*c*_*id*_) (i.e., ${\tilde{P}}_{\mathrm{id}}$) rather than the cluster-level prior probabilities [***V***_*k*_;***W***_*k*_]. Therefore, in the final AS calls, the model still allows each SNP to have its own AS configuration which may not necessarily be the same as the AS configurations of other SNPs from the same cluster.

Consistent with [38], in the two non-background AS patterns discovered here, proteins skewed toward the same direction did not always correspond to known protein-protein interactions. As pointed out by [38], this could happen as a result of allelic imbalances of different proteins being caused by a common underlying factor such as allelic difference in chromatin landscape. It could also reflect unknown protein-protein interactions. For iASeq specifically, there is a third reason, that is, multiple small patterns can be merged into a bigger probabilistic class as described before. For example, because of the use of probabilistic patterns, two patterns [SR,SR,NS,NS] and [NS,NS,SR,SR] may be merged into a single SNP class (e.g., ***V***_*k*_;***W***_*k*_=[(0*.*5,0*.*5,0*.*5,0*.*5);(0,0,0,0)]). As a result, only looking at the pattern represented by ***V***_*k*_;***W***_*k*_, one cannot tell the details of protein-protein interactions, such as these interactions only exist between datasets 1 and 2, or between 3 and 4, but not between the other pairs of datasets. What one can tell from this merged pattern is that, when the allelic imbalance occurs in these four datasets, they will be skewed toward the same direction, i.e., the reference allele in this example.

In summary, while the correlation patterns in iASeq provide some insights on the correlation of allelic imbalance among different datasets, one should not over-interpret them. The primary goal of these patterns is to describe the correlation structure in the data so that information from different datasets can be shared in a principled way to increase the power of statistical inference. This also points to an important difference between this study and previous studies that reported coordinated allele-specificity among multiple proteins. The previous studies only reported the correlation as a biological finding, but did not provide a statistical method to further utilize the correlation structure to improve the statistical inference. In contrast, iASeq provides a general and rigorous statistical method that utilizes the automatically discovered correlation patterns to increase the statistical power of AS detection. As such, it represents a novel development for the analysis of allele-specificity.

### Model, algorithm, and possible extensions

Unlike tools such as AlleleSeq which mainly focus on the preprocessing steps for the AS analysis (e.g., construction of diploid personal genome), iASeq is developed as a general model working downstream of the preprocessing pipelines. The input data for iASeq are the read counts in the format shown in Figure 1a. With this design, iASeq can be easily coupled with different data preprocessing protocols. For instance, some investigators may map their reads to a reference genome, while others may map their reads to a diploid personal genome. Both types of investigators can use iASeq to integrate information from multiple datasets once they obtained the allelic read counts.

In iASeq, we used an EM algorithm to find the posterior mode of parameters and carried out statistical inference accordingly. In principle, one may also use a full Bayesian approach and Markov Chain Monte Carlo (MCMC) to perform the posterior inference. However, since MCMC usually takes much longer to run for a big dataset and it is not easy for users to monitor convergence, we decided to use the posterior mode and EM-based approach in our implementation. For analyzing the GM12878 data with 94,519 SNPs, iASeq took 5 hours to run the EM algorithm to fit a single model with *K*=1 on a machine with 2.7 GHz CPU and 4Gb RAM. To fit a single model with *K*=10 on the same machine, the EM took 16 hours. Running the EM for all 10 *K*s between 1 and 10 on a single core took 4.6 days. However, when we run these 10 jobs in parallel on 10 cluster nodes, we were able to select the best model within 1 day. Therefore, running the algorithm on a single machine is a little time-consuming, but the computation time can be reduced by parallelization. Also, our analysis of GM12878 data indicates that the optimal *K* in that real data was 2. For a *K* not extremely large, even if running the full BIC selection on a single machine takes some time, it usually requires less than a week, which is acceptable compared to the time devoted to preparing samples and generating data.

In principle, the statistical model developed in iASeq may also be applied to analyze other types of AS events, such as ASE and ASM. In the future, we plan to improve the model by incorporating information from the spatial correlation among closely located SNPs. For example, for the ASE analysis, one may jointly model SNPs from the same gene, similar to [33].

### Implications on future studies

The analysis of AS events using the high-throughput sequencing data frequently faces the problem of low statistical power due to the limited amount of information available at heterozygote SNPs. One way to increase the power is to increase the sequencing depth for one data type (e.g., MYC ChIP-seq). An alternative approach is to spend the same amount of money to generate data for multiple different but related data types (e.g., ChIP-seq for MYC, H3K4me1, H3K4me3, etc.), each with a lower coverage. One can then integrate the multiple datasets to increase the statistical power of allele-specificity analysis. The merit of the second approach is that one can collect multiple different types of information which might be useful for other purposes (e.g., in addition to studying MYC binding using MYC ChIP-seq, one may couple H3K4me1 ChIP-seq data with DNA motif information to locate active enhancers and predict binding sites of other TFs in the genome). If the second approach is used in the study design, then iASeq will offer a flexible, powerful and scalable framework for better analyzing the AS events in the data. As ChIP-seq data continue to grow rapidly, this integrative approach will allow us to use the data more efficiently to characterize the allele-specificity.

## Conclusions

In summary, we have proposed a Bayesian hierarchical mixture model iASeq to integrate multiple ChIP-seq datasets for analyzing allele-specificity. The primary goal of iASeq is to increase the statistical power of AS detection, and it does so by taking the advantage of correlations among datasets. Since the correlation structure may not be known before the data analysis, iASeq learns it from the data automatically. Application of iASeq to the ENCODE GM12878 data shows that allelic imbalance of most analyzed TFs and HMs have strong preference to be skewed toward the same direction. Analysis of both the simulated and real data show the effectiveness of iASeq to improve detection of allele-specificity compared to single-dataset based methods.

## Abbreviations

AS: allele-specific; ASB: allele-specific binding, including both allele-specific TF binding and allele-specific histone modifications; ASE: allele-specific expression; ASM: allele-specific DNA methylation; AUC: area under receiver operating characteristic curve; EM: Expectation-Maximization algorithm; FDR: false discovery rate; HM: histone modification; NS: not allele-specific; ROC: receiver operating characteristic curve; SN: skewed to the non-reference allele; SR: skewed to the reference allele; TF: transcription factor.

## Competing interests

The authors declare that they have no competing interests.

## Authors’ contributions

Conceive study: HJ. Develop model: HJ, YW. Implementation: YW. Data collection: XL, QW. Data analysis: YW, XL. Write paper: HJ, YW, XL, QW. All authors read and approved the final manuscript.

## Supplementary Material

Additional file 1 **Supplemental Methods.** A PDF file including: data preprocessing procedures; method of moment estimation in the beta distribution; parameter choice for the Dirichlet prior; derivation of the EM algorithm for iASeq; Bayesian Information Criterion for choosing K; data generation procedure in simulation studies; single dataset based EM analysis. Click here for file

Additional file 2 **Table S1.** Description of all GM12878 ChIP-seq and RNA-seq studies. An excel file showing the name of TF and HM, the number of replicates for each dataset in GM12878 cells. Click here for file

Additional file 3 **Table S2.** Raw read count data for 94,519 analyzed SNPs. Click here for file

Additional file 4 **Supplemental comparison of defining allele-specific SNPs as SNPs that have RNA-seq exonic ASE SNPs in their 1kb neighborhood.** Supplemental Figure 3 — ROC curves for GM12878 using Caltech Exonic RNA-seq ASE SNPs as gold standard. Supplemental Figure 4 — ROC curves for GM12878 using Caltech autosomal exonic RNA-seq ASE SNPs as gold standard. Supplemental Figure 5 — ROC curves for GM12878 using Yale Exonic RNA-seq ASE SNPs as gold standard. Supplemental Figure 6 — ROC curves for GM12878 using Yale autosomal exonic RNA-seq ASE SNPs as gold standard. Supplemental Table 4 — Comparison of iASeq and AlleleSeq using Caltech RNA-seq exonic ASE SNPs as gold standard. Supplemental Table 5 — Comparison of iASeq and AlleleSeq using Yale RNA-seq exonic ASE SNPs as gold standard. Click here for file

Additional file 5 **Figure S1.** ROC curves for GM12878 using Yale Exonic RNA-seq ASE SNPs as gold standard. Click here for file

Additional file 6 **Figure S2.** ROC curves for GM12878 using Yale autosomal exonic RNA-seq ASE SNPs as gold standard. Click here for file

Additional file 7 **Table S3.** Comparison of iASeq and AlleleSeq using Yale RNA-seq exonic ASE SNPs as gold standard. Click here for file

Additional file 8 **Figure S7.** The ROC curves for comparison between AlleleSeq and iASeq. Click here for file

## References

[B1] Bell C Beck S Advances in the identification and analysis of allele specific expression Genome Med 2009 1 56 10.1186/gm56 19490587 PMC2689448

[B2] Graze R Novelo L Amin V Fear J Casella G Nuzhdin S McIntyre L Allelic imbalance in Drosophila hybrid heads: exons, isoforms, and evolution Mol Biol Evol 2012 29 1521 1532 10.1093/molbev/msr318 22319150 PMC3351786

[B3] Knight J Allele-specific gene expression uncovered Trends Genet 2004 20 113 116 10.1016/j.tig.2004.01.001 15049300

[B4] Chen R Mias G Li-Pook-Than J Jiang L Lam H Miriami E Karczewski K Hariharan M Dewey F Cheng Y Clark M Im H Habegger L Balasubramanian S O’Huallachain M Dudley J Hillenmeyer S Haraksingh R Sharon D Euskirchen G Lacroute P Bettinger K Boyle A Kasowski M Grubert F Seki S Garcia M Whirl-Carrillo M Gallardo M Blasco M Personal omics profiling reveals dynamic molecular and medical phenotypes Cell 2012 148 1293 1307 10.1016/j.cell.2012.02.009 22424236 PMC3341616

[B5] McDaniell R Lee B Song L Liu Z Boyle A Erdos M Scott L Morken M Kucera K Battenhouse A Keefe D Collins F Willard H Lieb J Furey T Crawford G Iyer V Birney E Heritable individual-specific and allele-specific chromatin signatures in humans Science 2010 328 235 239 10.1126/science.1184655 20299549 PMC2929018

[B6] Kasowski M Grubert F Heffelfinger C Hariharan M Asabere A Waszak S Habegger L Rozowsky J Shi M Urban A Hong M Karczewski K Huber W Weissman S Gerstein M Korbel J Snyder M Variation in transcription factor binding among humans Science 2010 328 232 235 10.1126/science.1183621 20299548 PMC2938768

[B7] Kerkel K Spadola A Yuan E Kosek J Jiang L Hod E Li K Murty V Schupf N Vilain E Morris M Haghighi F Tycko B Genomic surveys by methylation-sensitive SNP analysis identify sequence-dependent allele-specific DNA methylation Nat Genet 2008 40 904 908 10.1038/ng.174 18568024

[B8] Morley M Molony C Weber T Devlin J Ewens K Spielman R Cheung V Genetic analysis of genome-wide variation in human gene expression Nature 2004 430 743 747 10.1038/nature02797 15269782 PMC2966974

[B9] Schilling E El Chartouni C Rehli M Allele-specific DNA methylation in mouse strains is mainly determined by cis-acting sequences Genome Res 2009 19 2028 2035 10.1101/gr.095562.109 19687144 PMC2775599

[B10] Tycko B Allele-specific DNA methylation: beyond imprinting Hum Mol Genet 2010 19 R210 220 10.1093/hmg/ddq376 20855472 PMC2953749

[B11] Zhang K Li J Gao Y Egli D Xie B Deng J Li Z Lee J Aach J Leproust E Eggan K Church G Digital RNA allelotyping reveals tissue-specific and allele-specific gene expression in human Nat Methods 2009 6 613 618 10.1038/nmeth.1357 19620972 PMC2742772

[B12] Cui H Cruz-Correa M Giardiello F Hutcheon D Kafonek D Brandenburg S Wu Y He X Powe N Feinberg A Loss of IGF2 imprinting: a potential marker of colorectal cancer risk Science 2003 299 1753 1755 10.1126/science.1080902 12637750

[B13] Holt R Zhang Y Binia A Dixon A Vandiedonck C Cookson W Knight J Moffatt M Allele-specific transcription of the asthma-associated PHD finger protein 11 gene (PHF11) modulated by octamer-binding transcription factor 1 (Oct-1) J Allergy Clin Immunol 2011 127 1054 1062 e 1051–1052 10.1016/j.jaci.2010.12.015 21320718 PMC3955022

[B14] Heap G Yang J Downes K Healy B Hunt K Bockett N Franke L Dubois P Mein C Dobson R Albert T Rodesch M Clayton D Todd J van HD Plagnol V Genome-wide analysis of allelic expression imbalance in human primary cells by high-throughput transcriptome resequencing Human Mol Genet 2010 19 122 134 10.1093/hmg/ddp473 19825846 PMC2792152

[B15] Tuch B Laborde R Xu X Gu J Chung C Monighetti C Stanley S Olsen K Kasperbauer J Moore E Broomer A Tan R Brzoska P Muller M Siddiqui A Asmann Y Sun Y Kuersten S Barker M De LVF Smith D Tumor transcriptome sequencing reveals allelic expression imbalances associated with copy number alterations PLoS One 2010 5 2 e9317 10.1371/journal.pone.0009317 20174472 PMC2824832

[B16] Ben-David E Granot-Hershkovitz E Monderer-Rothkoff G Lerer E Levi S Yaari M Ebstein R Yirmiya N Shifman S Identification of a functional rare variant in autism using genome-wide screen for monoallelic expression Hum Mol Genet 2011 20 3632 3641 10.1093/hmg/ddr283 21680558

[B17] Lo H Wang Z Hu Y Yang H Gere S Buetow K Lee M Allelic variation in gene expression is common in the human genome Genome Res 2003 13 1855 1862 12902379 10.1101/gr.1006603 PMC403776

[B18] Palacios R Gazave E Goni J Piedrafita G Fernando O Navarro A Villoslada P Allele-specific gene expression is widespread across the genome and biological processes PLoS One 2009 4 1 e4150 10.1371/journal.pone.0004150 19127300 PMC2613524

[B19] Serre D Gurd S Ge B Sladek R Sinnett D Harmsen E Bibikova M Chudin E Barker D Dickinson T Fan J Hudson T Differential allelic expression in the human genome: a robust approach to identify genetic and epigenetic cis-acting mechanisms regulating gene expression PLoS Genet 2008 4 e1000006 10.1371/journal.pgen.1000006 18454203 PMC2265535

[B20] Chen P Feng S Joo J Jacobsen S Pellegrini M A comparative analysis of DNA methylation across human embryonic stem cell lines Genome Biol 2011 12 R62 10.1186/gb-2011-12-7-r62 21733148 PMC3218824

[B21] Montgomery S Sammeth M Gutierrez-Arcelus M Lach R Ingle C Nisbett J Guigo R Dermitzakis E Transcriptome genetics using second generation sequencing in a Caucasian population Nature 2010 464 773 U151 10.1038/nature08903 20220756 PMC3836232

[B22] Pickrell J Marioni J Pai A Degner J Engelhardt B Nkadori E Veyrieras J Stephens M Gilad Y Pritchard J Understanding mechanisms underlying human gene expression variation with RNA sequencing Nature 2010 464 768 772 10.1038/nature08872 20220758 PMC3089435

[B23] Ju Y Kim J Kim S Hong D Park H Shin J Lee S Lee W Kim S Yu S Park S Seo S Yun J Kim H Lee D Yavartanoo M Kang H Gokcumen O Govindaraju D Jung J Chong H Yang K Kim H Lee C Seo J Extensive genomic and transcriptional diversity identified through massively parallel DNA and RNA sequencing of eighteen Korean individuals Nat Genet 2011 43 745 U747 10.1038/ng.872 21725310

[B24] Tang F Barbacioru C Nordman E Bao S Lee C Wang X Tuch B Heard E Lao K Surani M Deterministic and stochastic allele specific gene expression in single mouse blastomeres PLoS One 2011 6 6 e21208 10.1371/journal.pone.0021208 21731673 PMC3121735

[B25] Barski A Cuddapah S Cui K Roh T Schones D Wang Z Wei G Chepelev I Zhao K High-resolution profiling of histone methylations in the human genome Cell 2007 129 823 837 10.1016/j.cell.2007.05.009 17512414

[B26] Johnson D Mortazavi A Myers R Wold B Genome-wide mapping of in vivo protein-DNA interactions Science 2007 316 1497 1502 10.1126/science.1141319 17540862

[B27] Mikkelsen T Ku M Jaffe D Issac B Lieberman E Giannoukos G Alvarez P Brockman W Kim T Koche R Lee W Mendenhall E O’Donovan A Presser A Russ C Xie X Meissner A Wernig M Jaenisch R Nusbaum C Lander E Bernstein B Genome-wide maps of chromatin state in pluripotent and lineage-committed cells Nature 2007 448 553 560 10.1038/nature06008 17603471 PMC2921165

[B28] Robertson G Hirst M Bainbridge M Bilenky M Zhao Y Zeng T Euskirchen G Bernier B Varhol R Delaney A Thiessen N Griffith O He A Marra M Snyder M Jones S Genome-wide profiles of STAT1 DNA association using chromatin immunoprecipitation and massively parallel sequencing Nat Methods 2007 4 651 657 10.1038/nmeth1068 17558387

[B29] Chen L Wu G Ji H hmChIP: a database and web server for exploring publicly available human and mouse ChIP-seq and ChIP-chip data Bioinformatics 2011 27 1447 1448 10.1093/bioinformatics/btr156 21450710 PMC3087956

[B30] hmChIP Database http://jilab.biostat.jhsph.edu/database/cgi-bin/hmChIP.pl

[B31] Degner J Marioni J Pai A Pickrell J Nkadori E Gilad Y Pritchard J Effect of read-mapping biases on detecting allele-specific expression from RNA-sequencing data Bioinformatics 2009 25 3207 3212 10.1093/bioinformatics/btp579 19808877 PMC2788925

[B32] Rozowsky J Abyzov A Wang J Alves P Raha D Harmanci A Leng J Bjornson R Kong Y Kitabayashi N Bhardwaj N Rubin M Snyder M Gerstein M AlleleSeq: analysis of allele-specific expression and binding in a network framework Mol Syst Biol 2011 7 522 21811232 10.1038/msb.2011.54 PMC3208341

[B33] Skelly D Johansson M Madeoy J Wakefield J Akey J A powerful and flexible statistical framework for testing hypotheses of allele-specific gene expression from RNA-seq data Genome Res 2011 21 1728 1737 10.1101/gr.119784.110 21873452 PMC3202289

[B34] Consortium EP The ENCODE (ENCyclopedia Of DNA Elements) Project Science 2004 306 636 640 15499007 10.1126/science.1105136

[B35] Bioconductor iASeq package. [http://www.bioconductor.org/packages/release/bioc/html/iASeq.html]

[B36] The ENCODE Project Consortium. An integrated encyclopedia of DNA elements in the human genome Nature 2012 489 57 74 10.1038/nature11247 22955616 PMC3439153

[B37] GM12878 genotype ftp://ftp-trace.ncbi.nih.gov/1000genomes/ftp/pilot_data/release/2010_07/trio/snps

[B38] Reddy T Gertz J Pauli F Kucera K Varley K Newberry K Marinov G Mortazavi A Williams B Song L Crawford G Wod B Willard H Myers R Effects of sequence variation on differential allelic transcription factor occupancy and gene expression Genome Res 2012 22 860 869 10.1101/gr.131201.111 22300769 PMC3337432

[B39] Kucera K Reddy T Pauli F Gertz J Logan J Myers R Willard H Allele-specific distribution of RNA polymerase II on female X chromosomes Hum Mol Genet 2011 20 3964 3973 10.1093/hmg/ddr315 21791549 PMC3177651

