# Supplementary material for: iASeq: integrative analysis of allele-specificity of protein-DNA interactions in multiple ChIP-seq datasets
Source: BMC Genomics. 2012 Nov 29;13:681. doi: 10.1186/1471-2164-13-681 (PMC3576346; doi:10.1186/1471-2164-13-681)
Supplement: Additional file 1 — Supplemental Methods. A PDF file including: data preprocessing procedures; method of moment estimation in the beta distribution; parameter choice for the Dirichlet prior; derivation of the EM algorithm for iASeq; Bayesian Information Criterion for choosing K; data generation procedure in simulation studies; single dataset based EM analysis. [file 1471-2164-13-681-S1.pdf]

Additional File 1 for iASeq: Supplemental Methods

S.1 Data preprocessing

S.1.1 Data collection

Both ChIP-seq and RNA-seq data for GM12878 cells were downloaded from the ENCODE Project [1]. The datasets used in the analysis are summarized in Additional File 2 Supplemental Table 1. For each ChIP-seq sample, we downloaded the FASTQ file and mapped the raw sequence reads to human genome (hg18) using MAQ (Version 0.7.1) with default parameters [2]. Uniquely mapped reads with the mapping quality score above 0 were extracted.

S.1.2 Heterozygote SNP collection, bias filtering and protein binding filtering

The genotype data for GM12878 was retrieved from the [3]. A total of 1,704,166 heterozygote SNPs were obtained. As shown by [4], in addition to the reference mapping bias (i.e., reference alleles are easier to be mapped back), certain SNPs have intrinsic biases toward one of the alleles. For these SNPs, genomic DNA extracted computationally from one allele (not necessarily the reference allele) is intrinsically easier to be mapped back compared to DNA extracted from the other allele. Using the method described in [4, 5], we identified and removed these 149,996 intrinsically biased SNPs. Next, we called ChIPseq peaks for each dataset using CisGenome [6] at 1% FDR level and retained only those heterozygous SNPs without intrinsic bias located in the protein binding regions in at least one dataset. After these two steps of filtering, 94,519 SNPs were included in our subsequent analysis.

S.1.3 Collection of exonic SNPs and ASE SNPs

The exonic ASE SNPs were used as one of our gold standards to evaluate iASeq and other methods. To determine which SNPs are exonic, we downloaded the hg18 Ensemble gene annotation file Homo_sapiens.NCBI36.54.gtf from [7]. Exonic SNPs were annotated using the exonic regions from

the gene annotation file. The exonic ASE SNPs were determined using RNA-seq. For a given RNA-seq dataset, the naive Bayes statistic was calculated for each exonic SNP. The top 400 exonic SNPs (6.61% of the total 6051 exonic SNPs) ranked based on the naive Bayes statistic were called as exonic ASE SNPs. Subsequently, in the analysis of ChIP-seq data, a SNP was claimed to be a true positive if there was an exonic ASE SNP within its Xkb neighborhood. Results for X=10kb are shown in the main manuscript and results for X=1kb are shown in Additional File 7. Both criteria gave similar results.

S.2 Method of moment for estimating parameters in the Beta distribution

For a Beta distribution $Beta(\alpha, \beta)$, the mean is $\alpha/(\alpha+\beta)$, and the variance is $\alpha\beta/(\alpha+\beta)^2(\alpha+\beta+1)$. For sample (d, j), each SNP has a p_{idj} which can be roughly estimated as $\hat{p}_{idj} = (x_{idj}+2*p^{(0)}_{dj0})/(n_{idj}+2)$. Here $p^{(0)}_{dj0} = \frac{1}{I^{(0)}_d}\sum_{i:n_{idj}\neq 0} x_{idj}/n_{idj}$, and $I^{(0)}_d$ is the number of SNPs in dataset d for which $n_{idj} \neq 0$. Let $p_{dj0} = \sum_i \hat{p}_{idj}/I$, and $v_{dj} = \frac{1}{I-1}\sum_i(p_{idj} - \hat{p}_{dj0})^2$. By matching p_{dj0} and v_{dj} to the theoretical mean and variance of a Beta distribution, we obtain

$$\hat{\alpha}_{dj} = p_{dj0} * [\frac{p_{dj0}(1-p_{dj0})}{v_{dj}} - 1] \tag{S.1}$$

$$\hat{\beta}_{dj} = (1-p_{dj0}) * [\frac{p_{dj0}(1-p_{dj0})}{v_{dj}} - 1] \tag{S.2}$$

In principle, one may develop a more sophisticated algorithm to estimate α and β by fitting beta-binomial distributions to $x_{idj}|n_{idj}$, but the computation will be more involved. Therefore we did not pursue this solution and instead used the simple method described above to approximately estimate α and β.

S.3 Parameter choice for the Dirichlet prior

Although $\eta = 1$ can specify an uniform prior and seems to be a natural choice, it will make the EM algorithm numerically unstable. This is because the EM searches for posterior mode and is implemented on log scale. The mode of a Dirichlet distribution $D(\eta_1, \cdots, \eta_M)$ for the m-th component is $(\eta_m - 1)/\sum_{l=1}^{M}(\eta_l - 1)$. It is not defined if all η_ms are equal to one. As a result, if $\eta = 1$ is used as the prior, and when the expectation of the counts $\sum_i \delta(a_i = k)$, $\sum_i \delta(a_i = k)b_{id}$ or $\sum_i \delta(a_i = k)c_{id}$ in the E-step of the algorithm is close to zero, then the algorithm can easily lose

its numerical stability due to issues such as $log(0)$ and ill-defined posterior mode. These issues can be avoided by using $\eta = 2$ which still imposes a relatively non-informative prior.

S.4 The EM algorithm used in iASeq

This section presents the EM algorithm used to search for posterior mode $(\hat{\boldsymbol{\pi}}, \hat{\boldsymbol{V}}, \hat{\boldsymbol{W}})$ of the distribution $Pr(\boldsymbol{\pi}, \boldsymbol{V}, \boldsymbol{W}|\boldsymbol{X}, \boldsymbol{N}) = \sum_{\boldsymbol{A},\boldsymbol{B},\boldsymbol{C}} Pr(\boldsymbol{A}, \boldsymbol{B}, \boldsymbol{C}, \boldsymbol{\pi}, \boldsymbol{V}, \boldsymbol{W}|\boldsymbol{X}, \boldsymbol{N})$. In the EM algorithm, $\boldsymbol{A}$, $\boldsymbol{B}$ and $\boldsymbol{C}$ are the missing data. The algorithm iterates between an E-step and an M-step.

In the E-step, one evaluates the Q-function $Q(\boldsymbol{\pi}, \boldsymbol{V}, \boldsymbol{W}|\hat{\boldsymbol{\pi}}^{\boldsymbol{old}}, \hat{\boldsymbol{V}}^{\boldsymbol{old}}, \hat{\boldsymbol{W}}^{\boldsymbol{old}})$ which is defined as $E_{old}[\ln Pr(\boldsymbol{A}, \boldsymbol{B}, \boldsymbol{C}, \boldsymbol{\pi}, \boldsymbol{V}, \boldsymbol{W}|\boldsymbol{X}, \boldsymbol{N})]$. Here the expectation is taken with respect to probability distribution $Pr(\boldsymbol{A}, \boldsymbol{B}, \boldsymbol{C}|\boldsymbol{X}, \boldsymbol{N}, \hat{\boldsymbol{\pi}}^{\boldsymbol{old}}, \hat{\boldsymbol{V}}^{\boldsymbol{old}}, \hat{\boldsymbol{W}}^{\boldsymbol{old}})$, abbreviated as $Pr_{old}(\boldsymbol{A}, \boldsymbol{B}, \boldsymbol{C})$, where $\hat{\boldsymbol{\pi}}^{\boldsymbol{old}}$, $\hat{\boldsymbol{V}}^{\boldsymbol{old}}$ and $\hat{\boldsymbol{W}}^{\boldsymbol{old}}$ are the parameter estimates obtained from the last iteration.

When we use $\eta = 2$ in the Dirichlet priors for $\boldsymbol{\pi}$, $\boldsymbol{V}$ and $\boldsymbol{W}$, we have

$$\begin{aligned}
& \ln Pr(\boldsymbol{A}, \boldsymbol{B}, \boldsymbol{C}, \boldsymbol{\pi}, \boldsymbol{V}, \boldsymbol{W}|\boldsymbol{X}, \boldsymbol{N}) \\
= & \sum_{i=1}^{I}\{\delta(a_i = 0)(\ln \pi_0 + \sum_{d=1}^{D} \ln L_{id0}) \\
+ & \sum_{k=1}^{K} \delta(a_i = k)[\ln \pi_k + \sum_{d=1}^{D} b_{id}(\ln v_{kd} + \ln L_{id1}) + \sum_{d=1}^{D} c_{id}(\ln w_{kd} + \ln L_{id2}) \\
+ & \sum_{d=1}^{D}(1 - b_{id} - c_{id})(\ln(1 - v_{kd} - w_{kd}) + \ln L_{id0})]\} \\
+ & \sum_{k=0}^{K} \ln \pi_k + \sum_{k=1}^{K}\sum_{d=1}^{D}[\ln v_{kd} + \ln w_{kd} + \ln(1 - v_{kd} - w_{kd})] \\
+ & \ constant
\end{aligned} \tag{S.3}$$

Therefore,

$$\begin{aligned}
& Q(\boldsymbol{\pi}, \boldsymbol{V}, \boldsymbol{W}|\hat{\boldsymbol{\pi}}^{\boldsymbol{old}}, \hat{\boldsymbol{V}}^{\boldsymbol{old}}, \hat{\boldsymbol{W}}^{\boldsymbol{old}}) \\
= & E_{old}[\ln Pr(\boldsymbol{A}, \boldsymbol{B}, \boldsymbol{C}, \boldsymbol{\pi}, \boldsymbol{V}, \boldsymbol{W}|\boldsymbol{X}, \boldsymbol{N})] \\
= & \sum_{k=0}^{K}\{\sum_{i=1}^{I} E_{old}[\delta(a_i = k)] + 1\} \ln \pi_k + \sum_{k=1}^{K}\sum_{d=1}^{D}\{(\sum_{i=1}^{I} E_{old}[\delta(a_i = k)b_{id}] + 1) \ln v_{kd} \\
+ & (\sum_{i=1}^{I} E_{old}[\delta(a_i = k)c_{id}] + 1) \ln w_{kd} + (\sum_{i=1}^{I} E_{old}[\delta(a_i = k)(1 - b_{id} - c_{id})] + 1) \ln(1 - v_{kd} - w_{kd})\} \\
+ & \ constant
\end{aligned} \tag{S.4}$$

In the M-step, one finds $\boldsymbol{\pi}$, $\boldsymbol{V}$ and $\boldsymbol{W}$ that maximize the Q-function $Q(\boldsymbol{\pi}, \boldsymbol{V}, \boldsymbol{W}|\hat{\boldsymbol{\pi}}^{\boldsymbol{old}}, \hat{\boldsymbol{V}}^{\boldsymbol{old}}, \hat{\boldsymbol{W}}^{\boldsymbol{old}})$. Denote them by $\hat{\boldsymbol{\pi}}^{\boldsymbol{new}}$, $\hat{\boldsymbol{V}}^{\boldsymbol{new}}$ and $\hat{\boldsymbol{W}}^{\boldsymbol{new}}$. These will give the new parameter estimates.

By solving

$$\frac{\partial Q(\boldsymbol{\pi}, \boldsymbol{V}, \boldsymbol{W}|\hat{\boldsymbol{\pi}}^{\boldsymbol{old}}, \hat{\boldsymbol{V}}^{\boldsymbol{old}}, \hat{\boldsymbol{W}}^{\boldsymbol{old}})}{\partial \pi_k} = 0 \tag{S.5}$$

$$\frac{\partial Q(\boldsymbol{\pi}, \boldsymbol{V}, \boldsymbol{W}|\hat{\boldsymbol{\pi}}^{\boldsymbol{old}}, \hat{\boldsymbol{V}}^{\boldsymbol{old}}, \hat{\boldsymbol{W}}^{\boldsymbol{old}})}{\partial v_{kd}} = 0 \tag{S.6}$$

$$\frac{\partial Q(\boldsymbol{\pi}, \boldsymbol{V}, \boldsymbol{W}|\hat{\boldsymbol{\pi}}^{\boldsymbol{old}}, \hat{\boldsymbol{V}}^{\boldsymbol{old}}, \hat{\boldsymbol{W}}^{\boldsymbol{old}})}{\partial w_{kd}} = 0 \tag{S.7}$$

We have

$$\hat{\pi}_k^{new} = \frac{\sum_{i=1}^{I} Pr_{old}(a_i = k) + 1}{I + K + 1} \tag{S.8}$$

$$\hat{v}_{kd}^{new} = \frac{\sum_{i=1}^{I} Pr_{old}(a_i = k, b_{id} = 1) + 1}{\sum_{i=1}^{I} Pr_{old}(a_i = k) + 3} \tag{S.9}$$

$$\hat{w}_{kd}^{new} = \frac{\sum_{i=1}^{I} Pr_{old}(a_i = k, c_{id} = 1) + 1}{\sum_{i=1}^{I} Pr_{old}(a_i = k) + 3} \tag{S.10}$$

In the formulas above, $Pr_{old}(a_i = k)$, $Pr_{old}(a_i = k, b_{id} = 1)$ and $Pr_{old}(a_i = k, c_{id} = 1)$ are computed as follows. To compute $Pr_{old}(a_i = k) = Pr(a_i = k|\boldsymbol{X}_i, \boldsymbol{N}_i, \hat{\boldsymbol{\pi}}^{\boldsymbol{old}}, \hat{\boldsymbol{V}}^{\boldsymbol{old}}, \hat{\boldsymbol{W}}^{\boldsymbol{old}})$, recall

$$Pr(a_i|\boldsymbol{X}_i, \boldsymbol{N}_i, \boldsymbol{\pi}, \boldsymbol{V}, \boldsymbol{W}) = Pr(\boldsymbol{X}_i, a_i|\boldsymbol{N}_i, \boldsymbol{\pi}, \boldsymbol{V}, \boldsymbol{W})/Pr(\boldsymbol{X}_i|\boldsymbol{N}_i, \boldsymbol{\pi}, \boldsymbol{V}, \boldsymbol{W}) \tag{S.11}$$

From Equation 5 in the main manuscript, we have

$$\begin{aligned} Pr(\boldsymbol{X}_i, a_i|\boldsymbol{N}_i, \boldsymbol{\pi}, \boldsymbol{V}, \boldsymbol{W}) &= \sum_{\boldsymbol{B}_i, \boldsymbol{C}_i} Pr(\boldsymbol{X}_i, a_i, \boldsymbol{B}_i, \boldsymbol{C}_i|\boldsymbol{N}_i, \boldsymbol{\pi}, \boldsymbol{V}, \boldsymbol{W}) \\ &= \{\pi_0 \prod_{d=1}^{D} L_{id0}\}^{\delta(a_i=0)} \prod_{k=1}^{K} \{\pi_k \prod_{d=1}^{D} [v_{kd}L_{id1} + w_{kd}L_{id2} + (1 - v_{kd} - w_{kd})L_{id0}]\}^{\delta(a_i=k)} \end{aligned} \tag{S.12}$$

and

$$\begin{aligned} Pr(\boldsymbol{X}_i|\boldsymbol{N}_i, \boldsymbol{\pi}, \boldsymbol{V}, \boldsymbol{W}) &= \sum_{a_i} Pr(\boldsymbol{X}_i, a_i|\boldsymbol{N}_i, \boldsymbol{\pi}, \boldsymbol{V}, \boldsymbol{W}) \\ &= \pi_0 \prod_{d=1}^{D} L_{id0} + \sum_{k=1}^{K} \{\pi_k \prod_{d=1}^{D} [v_{kd}L_{id1} + w_{kd}L_{id2} + (1 - v_{kd} - w_{kd})L_{id0}]\} \end{aligned} \tag{S.13}$$

Therefore, $Pr_{old}(a_i = k)$ can be computed by replacing $\boldsymbol{\pi}$, $\boldsymbol{V}$ and $\boldsymbol{W}$ by $\hat{\boldsymbol{\pi}}^{\boldsymbol{old}}$, $\hat{\boldsymbol{V}}^{\boldsymbol{old}}$ and $\hat{\boldsymbol{W}}^{\boldsymbol{old}}$.

$Pr_{old}(a_i = k, b_{id} = 1) = Pr_{old}(a_i = k)Pr_{old}(b_{id} = 1|a_i = k)$. $Pr_{old}(a_i = k)$ is computed as above. However,$Pr(b_{id}, c_{id}|a_i = k, \boldsymbol{X}_i, \boldsymbol{N}_i, \boldsymbol{\pi}, \boldsymbol{V}, \boldsymbol{W})$ can be computed as

$$\begin{aligned} \frac{Pr(b_{id}, c_{id}, \boldsymbol{X}_i|a_i = k, \boldsymbol{N}_i, \boldsymbol{\pi}, \boldsymbol{V}, \boldsymbol{W})}{Pr(\boldsymbol{X}_i|a_i = k, \boldsymbol{N}_i, \boldsymbol{\pi}, \boldsymbol{V}, \boldsymbol{W})} &= \frac{Pr(b_{id}, c_{id}, \boldsymbol{X}_{id}|a_i = k, \boldsymbol{N}_i, \boldsymbol{\pi}, \boldsymbol{V}, \boldsymbol{W})}{\sum_{b_{id}, c_{id}} Pr(b_{id}, c_{id}, \boldsymbol{X}_{id}|a_i = k, \boldsymbol{N}_i, \boldsymbol{\pi}, \boldsymbol{V}, \boldsymbol{W})} \\ &= \frac{[v_{kd}L_{id1}]^{b_{id}}[w_{kd}L_{id2}]^{c_{id}}[(1 - v_{kd} - w_{kd})L_{id0}]^{1-b_{id}-c_{id}}}{v_{kd}L_{id1} + w_{kd}L_{id2} + (1 - v_{kd} - w_{kd})L_{id0}} \end{aligned} \tag{S.14}$$

$Pr_{old}(b_{id} = 1|a_i = k)$ and $Pr_{old}(c_{id} = 1|a_i = k)$ can be obtained by plugging in $\hat{\boldsymbol{\pi}}^{\boldsymbol{old}}$, $\hat{\boldsymbol{V}}^{\boldsymbol{old}}$ and $\hat{\boldsymbol{W}}^{\boldsymbol{old}}$ into the formula above to replace $\boldsymbol{\pi}$, $\boldsymbol{V}$ and $\boldsymbol{W}$.

S.5 Bayesian Information Criterion (BIC) for choosing K

We compute BIC as

$$\begin{aligned} BIC(K) &= -2 * \ln\{\prod_{i=1}^{I} Pr(\boldsymbol{X}_i|\boldsymbol{N}_i, \boldsymbol{\pi}, \boldsymbol{V}, \boldsymbol{W})\} + (K + 2 * K * D) * \ln I \\ &= -2 * \sum_{i=1}^{I} \ln \left[\pi_0 \prod_{d=1}^{D} L_{id0} + \sum_{k=1}^{K} \{\pi_k \prod_{d=1}^{D} [v_{kd} L_{id1} + w_{kd} L_{id2} + (1 - v_{kd} - w_{kd}) L_{id0}]\} \right] \\ &\quad + K(2D + 1) \ln I \end{aligned} \tag{S.15}$$

We calculate BIC for different values of K, and choose the K with the smallest BIC. Here $K + 1$ is the number of classes. K is also the number of parameters in $\boldsymbol{\pi}$. $2KD$ is the number of parameters involved in $\boldsymbol{V}$ and $\boldsymbol{W}$. I is the SNP number. Strictly speaking, the data likelihood also involve terms $Pr(\boldsymbol{N}_i|\boldsymbol{\pi}, \boldsymbol{V}, \boldsymbol{W})$. However, based on our assumption, these terms do not depend on K, $\boldsymbol{\pi}$, $\boldsymbol{V}$ and $\boldsymbol{W}$, and can be reduced to $Pr(\boldsymbol{N}_i)$. They can be viewed as constants for the purpose of choosing the optimal K. We do not include them in the BIC computation.

S.6 Data generation in simulation studies

To simulate a ASB SNP i, we first sampled a SNP from the 8166 non-background SNPs in the real GM12878 data. Here the non-background SNPs in the real data were determined by iASeq using $Pr(a_i = 0|\boldsymbol{X}_i, \boldsymbol{N}_i, \boldsymbol{\pi}, \boldsymbol{V}, \boldsymbol{W}) < 0.5$ as cutoff. Additionally, we also sampled a SNP from the 86,353 background SNPs in the real GM12878 data. Next, with these two real SNPs in hand, we went through each dataset d to generate the read counts for the simulated SNP. If $[b_{id}, c_{id}] = [0, 0]$, then we used the background SNP's read count data (x_{idj}, n_{idj}) in sample (d, j) to serve as the data of the simulated SNP in dataset d sample j. If $[b_{id}, c_{id}] = [1, 0]$, then we used the non-background SNP's read count data to simulate read counts as follows. For each replicate sample j in dataset d, we obtained the observed total read count n_{idj} for the non-background SNP. We then randomly drew a number p_{idj} from $U[p_{dj0}, 1]$, where p_{dj0} is the mean of $\frac{x_{idj}}{n_{idj}}$ over all background SNPs in the same sample (d, j). Subsequently, we simulated x_{idj} from a binomial distribution $Bin(n_{idj}, p_{idj})$ to serve as the simulated SNP's data in dataset d and sample j. If $[b_{id}, c_{id}] = [0, 1]$, we applied a similar procedure but the p_{idj} was drawn from $U[0, p_{dj0}]$.

S.7 The single dataset based EM analysis

This approach analyzes each dataset separately. Let $\boldsymbol{X}^d = (\boldsymbol{X}_{1d}, \cdots, \boldsymbol{X}_{Id})$ and $\boldsymbol{N}^d = (\boldsymbol{N}_{1d}, \cdots, \boldsymbol{N}_{Id})$ be the data from dataset d. We assumed that in each dataset d, a SNP i can be SR ($b_{id} = 1$), SN ($c_{id} = 1$) or NS ($b_{id} = 0$ and $c_{id} = 0$) with probability $(v_d, w_d, 1 - v_d - w_d)$. Let $\boldsymbol{B}^d = (b_{1d}, \cdots, b_{Id})$ and $\boldsymbol{C}^d = (c_{1d}, \cdots, c_{Id})$ be the ensemble of all ASB indicators in dataset d. Adopting the same distributional assumption as in Equations 1-3 in the main mainuscript, the complete data likelihood can be derived as:

$$\begin{aligned} Pr(\boldsymbol{X}^d, \boldsymbol{N}^d, \boldsymbol{B}^d, \boldsymbol{C}^d | v_d, w_d) &= Pr(\boldsymbol{N}^d) Pr(\boldsymbol{X}^d, \boldsymbol{B}^d, \boldsymbol{C}^d | \boldsymbol{N}^d, v_d, w_d) \\ &= Pr(\boldsymbol{N}^d) \prod_{i=1}^{I} \{[v_d L_{id1}]^{b_{id}} [w_d L_{id2}]^{c_{id}} [(1 - v_d - w_d) L_{id0}]^{(1 - b_{id} - c_{id})}\} \end{aligned} \tag{S.16}$$

By imposing a Dirichlet prior $D(2, 2, 2)$ on $(v_d, w_d, 1 - v_d - w_d)$, we obtain the posterior distribution of the unknown parameters and missing indicators:

$$Pr(\boldsymbol{B}^d, \boldsymbol{C}^d, v_d, w_d | \boldsymbol{X}^d, \boldsymbol{N}^d) \propto \prod_{i=1}^{I} \{[v_d L_{id1}]^{b_{id}} [w_d L_{id2}]^{c_{id}} [(1 - v_d - w_d) L_{id0}]^{(1 - b_{id} - c_{id})}\} v_d w_d (1 - v_d - w_d) \tag{S.17}$$

An EM algorithm can be similarly derived as in iASeq to estimate the parameters v_d and w_d by searching for the posterior mode of $Pr(v_d, w_d | \boldsymbol{X}^d, \boldsymbol{N}^d)$.

In the E-step, we compute the Q-function $Q(v_d, w_d | \hat{v}_d^{old}, \hat{w}_d^{old})$. Since

$$\begin{aligned} \ln Pr(\boldsymbol{B}^d, \boldsymbol{C}^d, v_d, w_d | \boldsymbol{X}^d, \boldsymbol{N}^d) &= \sum_{i=1}^{I} \{b_{id}[\ln v_d + \ln L_{id1}] + c_{id}[\ln w_d + \ln L_{id2}] \\ &+ (1 - b_{id} - c_{id})[\ln(1 - v_d - w_d) + \ln L_{id0}]\} + \ln v_d \\ &+ \ln w_d + \ln(1 - v_d - w_d) + constant \end{aligned} \tag{S.18}$$

We have

$$\begin{aligned} Q(v_d, w_d | \hat{v}_d^{old}, \hat{w}_d^{old}) &= E_{old}[\ln Pr(\boldsymbol{B}^d, \boldsymbol{C}^d, v_d, w_d | \boldsymbol{X}^d, \boldsymbol{N}^d)] \\ &= \{\sum_{i=1}^{I} E_{old}(b_{id}) + 1\} \ln v_d + \{\sum_{i=1}^{I} E_{old}(c_{id}) + 1\} \ln w_d \\ &+ \{\sum_{i=1}^{I} E_{old}(1 - b_{id} - c_{id}) + 1\} \ln(1 - v_d - w_d) + constant \end{aligned} \tag{S.19}$$

In the M-step, we find v_d and w_d that maximize $Q(v_d, w_d | \hat{v}_d^{old}, \hat{w}_d^{old})$. By solving

$$\frac{\partial Q(v_d, w_d | \hat{v}_d^{old}, \hat{w}_d^{old})}{\partial v_d} = 0 \tag{S.20}$$

$$\frac{\partial Q(v_d, w_d | \hat{v}_d^{old}, \hat{w}_d^{old})}{\partial w_d} = 0 \tag{S.21}$$

We obtain

$$\hat{v}_d^{new} = \frac{\sum_{i=1}^{I} Pr_{old}(b_{id} = 1) + 1}{I + 3} \tag{S.22}$$

$$\hat{w}_d^{new} = \frac{\sum_{i=1}^{I} Pr_{old}(c_{id} = 1) + 1}{I + 3} \tag{S.23}$$

Here

$$\begin{aligned} Pr_{old}(b_{id} = 1) &= Pr(b_{id} = 1 | \boldsymbol{X}_{id}, \boldsymbol{N}_{id}, \hat{v}_d^{old}, \hat{w}_d^{old}) = \frac{Pr(b_{id} = 1, \boldsymbol{X}_{id} | \boldsymbol{N}_{id}, \hat{v}_d^{old}, \hat{w}_d^{old})}{Pr(\boldsymbol{X}_{id} | \boldsymbol{N}_{id}, \hat{v}_d^{old}, \hat{w}_d^{old})} \\ &= \frac{\hat{v}_d^{old} L_{id1}}{\hat{v}_d^{old} L_{id1} + \hat{w}_d^{old} L_{id2} + (1 - \hat{v}_d^{old} - \hat{w}_d^{old}) L_{id0}} \end{aligned} \tag{S.24}$$

$$\begin{aligned} Pr_{old}(c_{id} = 1) &= Pr(c_{id} = 1 | \boldsymbol{X}_{id}, \boldsymbol{N}_{id}, \hat{v}_d^{old}, \hat{w}_d^{old}) = \frac{Pr(c_{id} = 1, \boldsymbol{X}_{id} | \boldsymbol{N}_{id}, \hat{v}_d^{old}, \hat{w}_d^{old})}{Pr(\boldsymbol{X}_{id} | \boldsymbol{N}_{id}, \hat{v}_d^{old}, \hat{w}_d^{old})} \\ &= \frac{\hat{w}_d^{old} L_{id2}}{\hat{v}_d^{old} L_{id1} + \hat{w}_d^{old} L_{id2} + (1 - \hat{v}_d^{old} - \hat{w}_d^{old}) L_{id0}} \end{aligned} \tag{S.25}$$

Using the posterior mode, one can similarly compute $Pr(b_{id}, c_{id} | \boldsymbol{X}^d, \boldsymbol{N}^d, v_d, w_d)$ and $\tilde{P}_{id}$ to detect and rank AS SNPs.

References

[1] **The ENCODE Project Consortium. An integrated encyclopedia of DNA elements in the human genome**. *Nature* 2012, **489**:57–74.

[2] Li H, Ruan J, Durbin R: **Mapping short DNA sequencing reads and calling variants using mapping quality scores**. *Genome Res* 2008, **18**:1851–1858.

[3] **GM12878 genotype** [`ftp://ftp-trace.ncbi.nih.gov/1000genomes/ftp/pilot_data/release/2010_07/trio/snps`].

[4] Degner J, Marioni J, Pai A, Pickrell J, Nkadori E, Gilad Y, Pritchard J: **Effect of read-mapping biases on detecting allele-specific expression from RNA-sequencing data**. *Bioinformatics* 2009, **25**:3207–3212.

[5] Pickrell J, Marioni J, Pai A, Degner J, Engelhardt B, Nkadori E, Veyrieras J, Stephens M, Gilad Y, Pritchard J: **Understanding mechanisms underlying human gene expression variation with RNA sequencing**. *Nature* 2010, **464**:768–772.

[6] Ji H, Jiang H, Ma W, Johnson D, Myers R, Wong W: **An integrated software system for analyzing ChIP-chip and ChIP-seq data**. *Nature Biotechnology* 2008, :1293–1300.

[7] **hg18 Ensemble gene annotation** [ftp://ftp.ensembl.org/pub/release-54/gtf/homo_sapiens/].
