# Supplementary material for: iASeq: integrative analysis of allele-specificity of protein-DNA interactions in multiple ChIP-seq datasets
Source: BMC Genomics. 2012 Nov 29;13:681. doi: 10.1186/1471-2164-13-681 (PMC3576346; doi:10.1186/1471-2164-13-681)
Supplement: Additional file 4 — Supplemental comparison of defining allele-specific SNPs as SNPs that have RNA-seq exonic ASE SNPs in their 1kb neighborhood. Supplemental Figure 3 — ROC curves for GM12878 using Caltech Exonic RNA-seq ASE SNPs as gold standard. Supplemental Figure 4 — ROC curves for GM12878 using Caltech autosomal exonic RNA-seq ASE SNPs as gold standard. Supplemental Figure 5 — ROC curves for GM12878 using Yale Exonic RNA-seq ASE SNPs as gold standard. Supplemental Figure 6 — ROC curves for GM12878 using Yale autosomal exonic RNA-seq ASE SNPs as gold standard. Supplemental Table 4 — Comparison of iASeq and AlleleSeq using Caltech RNA-seq exonic ASE SNPs as gold standard. Supplemental Table 5 — Comparison of iASeq and AlleleSeq using Yale RNA-seq exonic ASE SNPs as gold standard. [file 1471-2164-13-681-S4.pdf]

Additional File 4 for iASeq: supplemental comparison of defining allele-specific SNPs as SNPs that have ≥ 1 RNA-seq exonic ASE SNPs in their 1kb neighborhood

Supplementary Figure 3: The ROC curves in GM12878 data using Caltech RNA-seq ASE SNPs as gold standard. We plot $TP_d(q)$, the number of true allele-specific SNPs among the top q ranked SNPs in dataset d, against the rank cutoff q for each method. The true allele-specific SNPs are defined as SNPs that have ≥ 1 RNA-seq exonic ASE SNPs in their 1kb neighborhood. (a)-(g) Results in 7 representative datasets. (h) In each dataset, we computed the area under the ROC curve (AUC) using the 2000 top ranked SNPs for each method. dAUC, the proportion of improvement of AUC brought by iASeq over the best AUC from the single-dataset based methods, was computed for each dataset. The distribution of dAUC in all 40 datasets is shown.

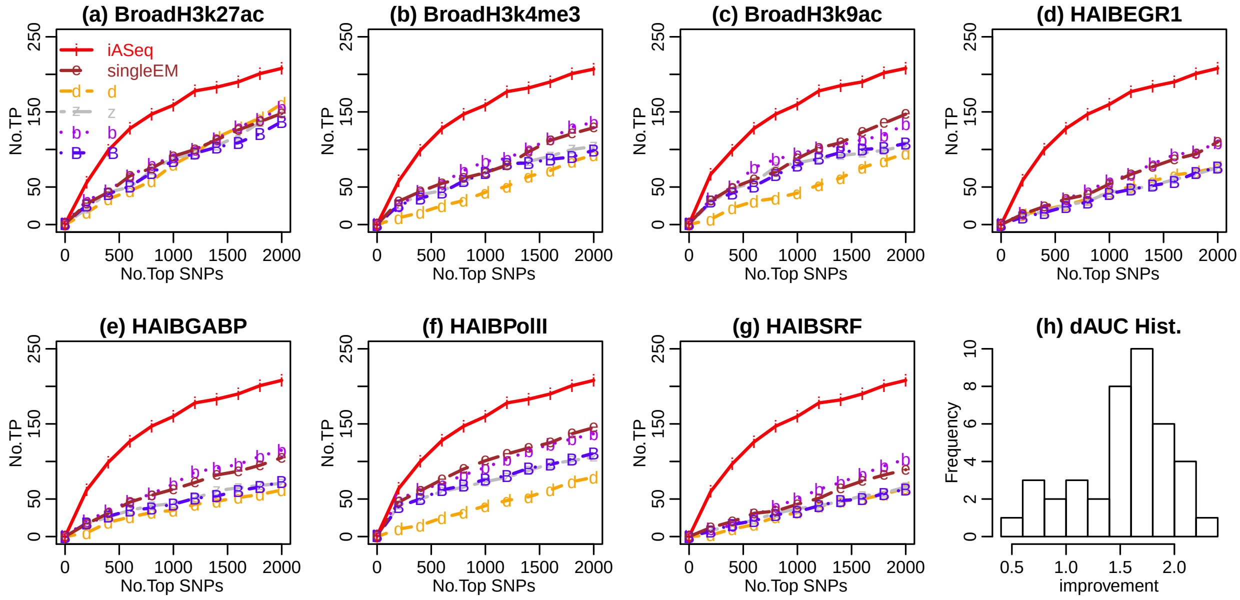

Supplementary Figure 4: The ROC curves in GM12878 data using Caltech RNA-seq autosomal ASE SNPs as gold standard We plot $TP_d(q)$, the number of true allele-specific SNPs among the top q ranked autosomal SNPs in dataset d, against the rank cutoff q for each method. The true allele-specific SNPs are defined as autosomal SNPs that have ≥ 1 RNA-seq exonic ASE SNPs in their 1kb neighborhood. (a)-(g) Results in 7 representative datasets. (h) In each dataset, we computed the area under the ROC curve (AUC) using the 2000 top ranked SNPs for each method. dAUC, the proportion of improvement of AUC brought by iASeq over the best AUC from the single-dataset based methods, was computed for each dataset. The distribution of dAUC in all 40 datasets is shown.

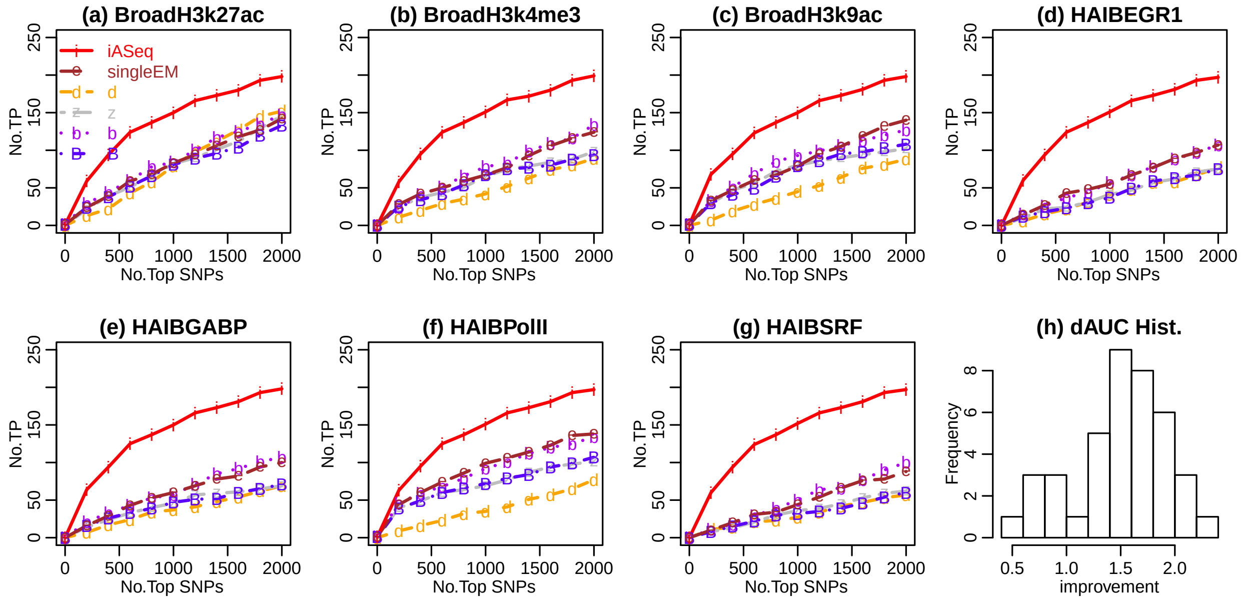

Supplementary Figure 5. ROC curves for GM12878 using Yale Exonic RNA-seq ASE SNPs as gold standard. We plot $TP_d(q)$, the number of true allele-specific SNPs among the top q ranked SNPs in dataset d, against the rank cutoff q for each method. The true allele-specific SNPs are defined as SNPs that have ≥ 1 RNA-seq exonic ASE SNPs in their 1kb neighborhood. (a)-(g) Results in 7 representative datasets. (h) In each dataset, we computed the area under the ROC curve (AUC) using the 2000 top ranked SNPs for each method. dAUC, the proportion of improvement of AUC brought by iASeq over the best AUC from the single-dataset based methods, was computed for each dataset. The distribution of dAUC in all 40 datasets is shown.

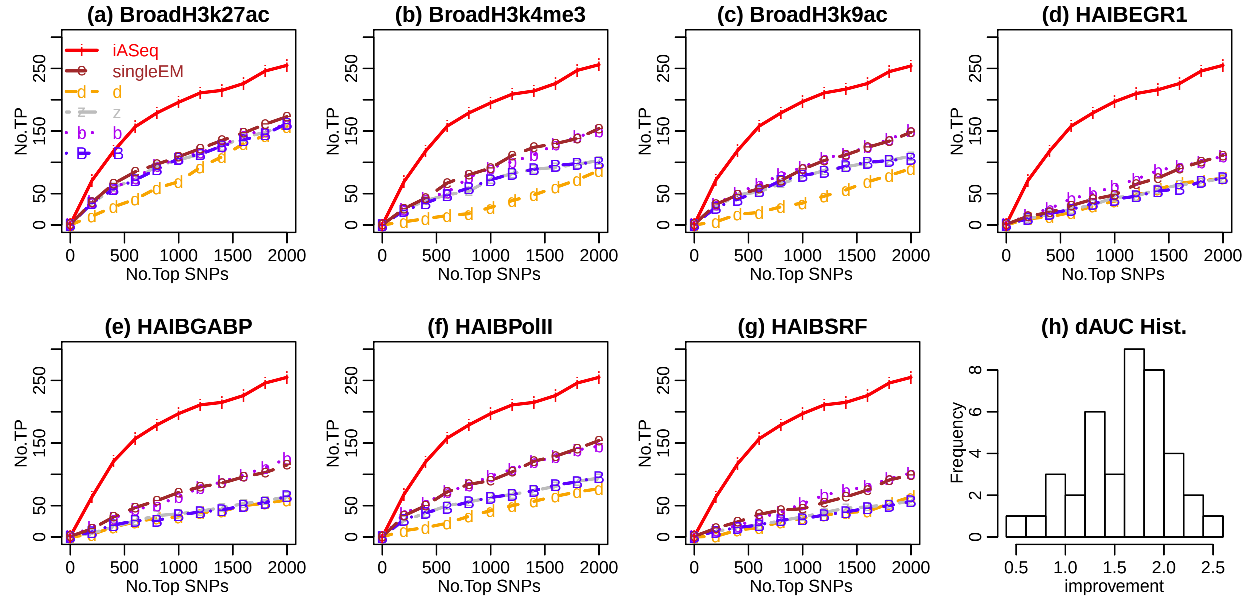

Supplemental Figure 6. ROC curves for GM12878 using Yale autosomal exonic RNA-seq ASE SNPs as gold standard. We plot $TP_d(q)$, the number of true allele-specific SNPs among the top q ranked autosomal SNPs in dataset d, against the rank cutoff q for each method. The true allele-specific SNPs are defined as autosomal SNPs that have ≥ 1 RNA-seq exonic ASE SNPs in their 1kb neighborhood. (a)-(g) Results in 7 representative datasets. (h) In each dataset, we computed the area under the ROC curve (AUC) using the 2000 top ranked SNPs for each method. dAUC, the proportion of improvement of AUC brought by iASeq over the best AUC from the single-dataset based methods, was computed for each dataset. The distribution of dAUC in all 40 datasets is shown.

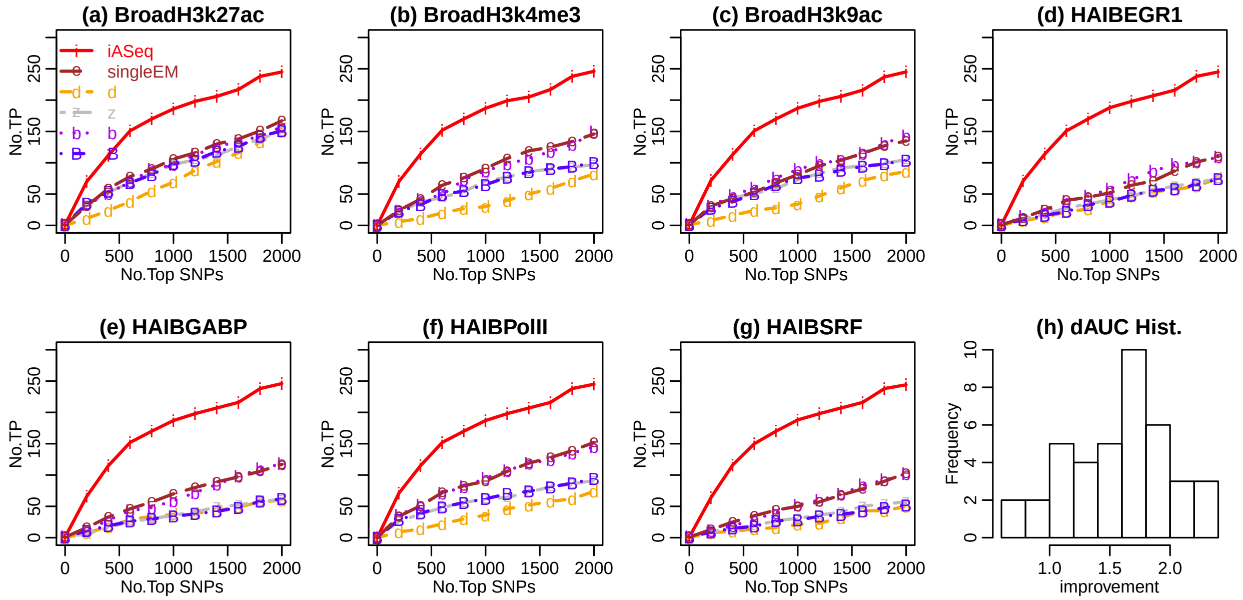

Supplementary Table 4: Comparison of iASeq and AlleleSeq using Caltech RNA-seq exonic ASE SNPs as gold standard. Column 1: TF name. Column 2: T_d is the number of AlleleSeq reported ASB SNPs. Columns 3-4: the number of non-pseudoautosomal region X chromosome SNPs among the top T_d allele-specific SNPs reported by AlleleSeq and iASeq. Column 5: T_d is the number of AlleleSeq reported ASB SNPs that had an exonic SNP within their 1kb neighborhood. Columns 6-7 show among the top T_d allele-specific SNPs reported by AlleleSeq and iASeq, how many SNPs had ≥ 1 exonic ASE SNP in their 1kb neighborhood according to the Caltech RNA-seq experiment. Column 8: T_d is the number of AlleleSeq reported autosomal ASB SNPs that had an exonic SNP within their 1kb neighborhood. Columns 9-10 show among the top T_d autosomal allele-specific SNPs reported by AlleleSeq and iASeq, how many SNPs had ≥ 1 exonic ASE SNP in their 1kb neighborhood according to the Caltech RNA-seq experiment.

Gold standard	ChrX			All Caltech ASE exonic SNPs			Autosomal Caltech ASE exonic SNPs		
TF	T_d	AlleleSeq	iASeq	T_d	AlleleSeq	iASeq	T_d	AlleleSeq	iASeq
YaleCFOS	41	3	4	3	0	1	3	0	1
YaleMYC	122	9	22	15	3	4	14	3	4
YaleJUND	289	13	31	13	2	6	12	2	6
YaleMAX	105	3	18	6	2	0	6	2	0
YalePolIII	25	2	2	0	0	0	0	0	0

Supplementary Table 5: Comparison of iASeq and AlleleSeq using Yale RNA-seq exonic ASE SNPs as gold standard. Column 1: TF; Column 2: T_d is the number of AlleleSeq reported ASB SNPs that had an exonic SNP within their 1kb neighborhood. Columns 3-4 show among the top T_d allele-specific SNPs reported by AlleleSeq and iASeq, how many SNPs had ≥ 1 exonic ASE SNP in their 1kb neighborhood according to the Yale RNA-seq experiment. Column 5: T_d is the number of AlleleSeq reported autosomal ASB SNPs that had an exonic SNP within their 1kb neighborhood. Columns 6-7 show among the top T_d autosomal allele-specific SNPs reported by AlleleSeq and iASeq, how many SNPs had ≥ 1 exonic ASE SNP in their 1kb neighborhood according to the Yale RNA-seq experiment.

Gold standard	All Yale ASE exonic SNPs			Autosomal Yale ASE exonic SNPs		
TF	T_d	Alleleseq	iASeq	T_d	Alleleseq	iASeq
YaleCFOS	3	0	2	3	0	2
YaleMYC	15	5	5	14	5	5
YaleJUND	13	1	4	12	1	4
YaleMAX	6	5	3	6	5	3
YalePolIII	0	0	0	0	0	0
