# Supplementary material for: iASeq: integrative analysis of allele-specificity of protein-DNA interactions in multiple ChIP-seq datasets
Source: BMC Genomics. 2012 Nov 29;13:681. doi: 10.1186/1471-2164-13-681 (PMC3576346; doi:10.1186/1471-2164-13-681)

Additional File 5 for iASeq

Supplementary Figure 1. ROC curves for GM12878 using Yale Exonic RNA-seq ASE SNPs as gold standard. We plot $TP_d(q)$, the number of true allele-specific SNPs among the top q ranked SNPs in dataset d, against the rank cutoff q for each method. The true allele-specific SNPs are defined as SNPs that have ≥ 1 RNA-seq exonic ASE SNPs in their 10kb neighborhood. (a)-(g) Results in 7 representative datasets. (h) In each dataset, we computed the area under the ROC curve (AUC) using the 2000 top ranked SNPs for each method. dAUC, the proportion of improvement of AUC brought by iASeq over the best AUC from the single-dataset based methods, was computed for each dataset. The distribution of dAUC in all 40 datasets is shown.

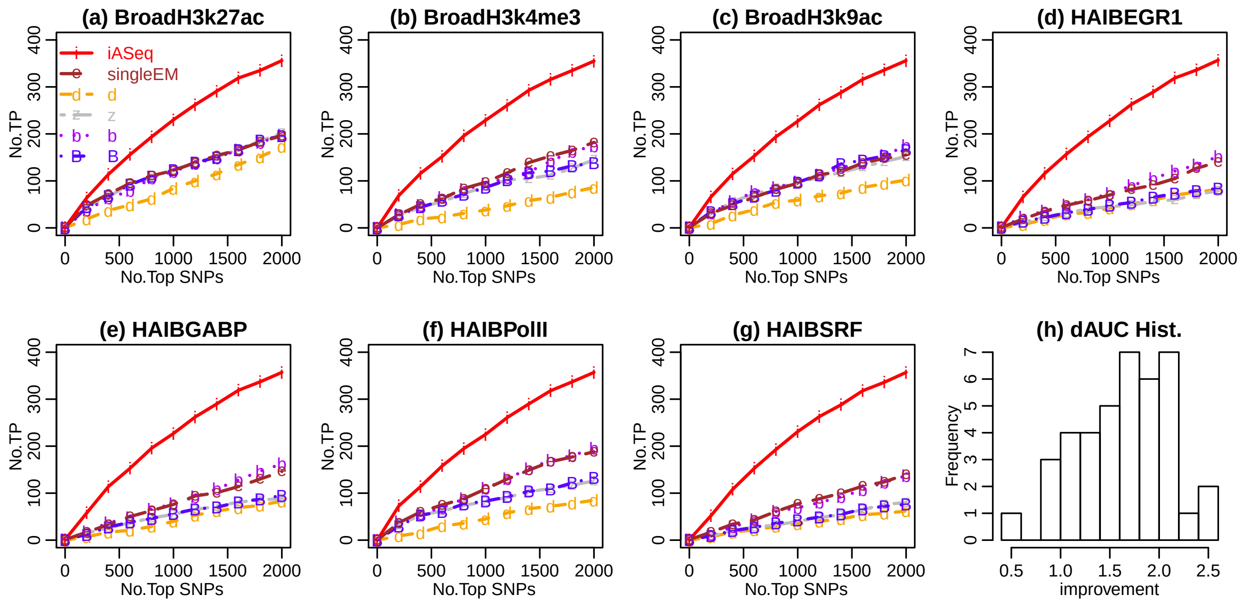

Supplement: Additional file 5 — Figure S1. ROC curves for GM12878 using Yale Exonic RNA-seq ASE SNPs as gold standard. [file 1471-2164-13-681-S5.pdf]
