# Supplementary material for: iASeq: integrative analysis of allele-specificity of protein-DNA interactions in multiple ChIP-seq datasets
Source: BMC Genomics. 2012 Nov 29;13:681. doi: 10.1186/1471-2164-13-681 (PMC3576346; doi:10.1186/1471-2164-13-681)
Supplement: Additional file 7 — Table S3. Comparison of iASeq and AlleleSeq using Yale RNA-seq exonic ASE SNPs as gold standard. [file 1471-2164-13-681-S7.pdf]

Additional File 7 for iASeq

Supplementary Table 3: Comparison of iASeq and AlleleSeq using Yale RNA-seq exonic ASE SNPs as gold standard. Column 1: TF; Column 2: T_d is the number of AlleleSeq reported ASB SNPs that had an exonic SNP within their 10kb neighborhood. Columns 3-4 show among the top T_d allele-specific SNPs reported by AlleleSeq and iASeq, how many SNPs had $\geq$ 1 exonic ASE SNP in their 10kb neighborhood according to the Yale RNA-seq experiment. Column 5: T_d is the number of AlleleSeq reported autosomal ASB SNPs that had an exonic SNP within their 10kb neighborhood. Columns 6-7 show among the top T_d autosomal allele-specific SNPs reported by AlleleSeq and iASeq, how many SNPs had $\geq$ 1 exonic ASE SNP in their 10kb neighborhood according to the Yale RNA-seq experiment.

Gold standard	All Yale ASE exonic SNPs			Autosomal Yale ASE exonic SNPs		
TF	T_d	Alleleseq	iASeq	T_d	Alleleseq	iASeq
YaleCFOS	9	3	3	9	3	3
YaleMYC	39	10	13	38	10	13
YaleJUND	24	5	6	23	5	6
YaleMAX	18	6	5	18	6	5
YalePolIII	0	0	0	0	0	0
