# Supplementary material for: iASeq: integrative analysis of allele-specificity of protein-DNA interactions in multiple ChIP-seq datasets
Source: BMC Genomics. 2012 Nov 29;13:681. doi: 10.1186/1471-2164-13-681 (PMC3576346; doi:10.1186/1471-2164-13-681)

Additional File 8 for iASeq

Supplementary Figure 7: The ROC curves for comparison between AlleleSeq and iASeq. We plot $TP_d(q)$, the number of true positive (TP) SNPs among the top q ranked SNPs in dataset d, against the rank cutoff q for both methods. We only show cases where at least 10 TPs were identified by either method to present robust results. And therefore, when using RNA-seq to benchmark the performance, only results where TPs were defined as SNPs with exonic ASE SNPs existing in their 10 kb neighborhood are shown below. As shown in Additional File 7 Supplemental tables 4-5, neither method identified more than 10 TPs when TPs were defined as SNPs with exonic ASE SNPs existing in their 1 kb neighborhood and therefore no ROC curves shown below. (a)-(c) True positives were defined as non-pseudoautosomal region X chromosome SNPs for YaleMYC, YaleJUND, YaleMAX. (d) The true allele-specific SNPs were defined as SNPs that have ≥ 1 Caltech RNA-seq exonic ASE SNPs in their 10kb neighborhood. (e) The true allele-specific SNPs are defined as autosomal SNPs that have ≥ 1 Caltech RNA-seq exonic ASE SNPs in their 10kb neighborhood.(f) The true allele-specific SNPs were defined as SNPs that have ≥ 1 Yale RNA-seq exonic ASE SNPs in their 10kb neighborhood. (g) The true allele-specific SNPs are defined as autosomal SNPs that have ≥ 1 Yale RNA-seq exonic ASE SNPs in their 10kb neighborhood.

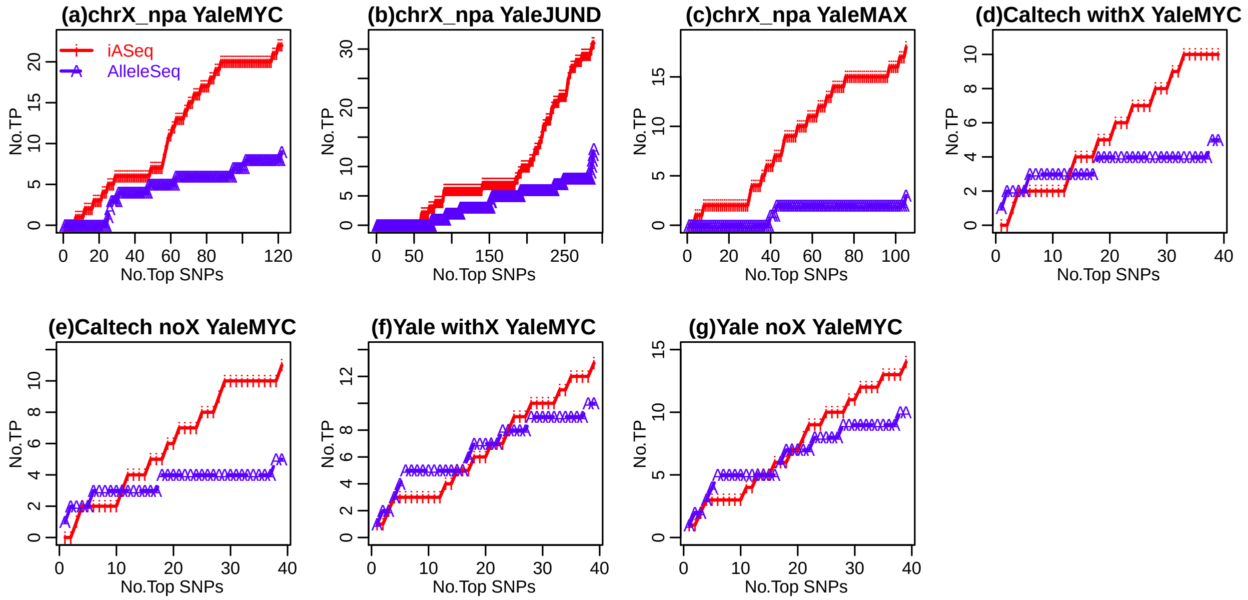

Supplement: Additional file 8 — Figure S7. The ROC curves for comparison between AlleleSeq and iASeq. [file 1471-2164-13-681-S8.pdf]
